# Distinct responses of newly identified monocyte subsets to advanced gastrointestinal cancer and COVID-19

**DOI:** 10.3389/fimmu.2022.967737

**Published:** 2022-10-03

**Authors:** Alessandra Rigamonti, Alessandra Castagna, Marika Viatore, Federico Simone Colombo, Sara Terzoli, Clelia Peano, Federica Marchesi, Massimo Locati

**Affiliations:** ^1^ Department of Immunology and Inflammation, IRCCS Humanitas Research Hospital, Milan, Italy; ^2^ Department of Medical Biotechnology and Translational Medicine, University of Milan, Milan, Italy; ^3^ Flow Cytometry Core, IRCCS Humanitas Research Hospital, Milan, Italy; ^4^ Laboratory of Clinical and Experimental Immunology, IRCCS Humanitas Research Hospital, Milan, Italy; ^5^ Genomic Unit, IRCCS Humanitas Research Hospital, Milan, Italy; ^6^ Institute of Genetic and Biomedical Research, UoS of Milan, National Research Council, Milan, Italy

**Keywords:** monocyte, single-cell transcriptome, machine learning, cancer, immunotherapy, COVID-19

## Abstract

Monocytes are critical cells of the immune system but their role as effectors is relatively poorly understood, as they have long been considered only as precursors of tissue macrophages or dendritic cells. Moreover, it is known that this cell type is heterogeneous, but our understanding of this aspect is limited to the broad classification in classical/intermediate/non-classical monocytes, commonly based on their expression of only two markers, i.e. CD14 and CD16. We deeply dissected the heterogeneity of human circulating monocytes in healthy donors by transcriptomic analysis at single-cell level and identified 9 distinct monocyte populations characterized each by a profile suggestive of specialized functions. The classical monocyte subset in fact included five distinct populations, each enriched for transcriptomic gene sets related to either inflammatory, neutrophil-like, interferon-related, and platelet-related pathways. Non-classical monocytes included two distinct populations, one of which marked specifically by elevated expression levels of complement components. Intermediate monocytes were not further divided in our analysis and were characterized by high levels of human leukocyte antigen (HLA) genes. Finally, we identified one cluster included in both classical and non-classical monocytes, characterized by a strong cytotoxic signature. These findings provided the rationale to exploit the relevance of newly identified monocyte populations in disease evolution. A machine learning approach was developed and applied to two single-cell transcriptome public datasets, from gastrointestinal cancer and Coronavirus disease 2019 (COVID-19) patients. The dissection of these datasets through our classification revealed that patients with advanced cancers showed a selective increase in monocytes enriched in platelet-related pathways. Of note, the signature associated with this population correlated with worse prognosis in gastric cancer patients. Conversely, after immunotherapy, the most activated population was composed of interferon-related monocytes, consistent with an upregulation in interferon-related genes in responder patients compared to non-responders. In COVID-19 patients we confirmed a global activated phenotype of the entire monocyte compartment, but our classification revealed that only cytotoxic monocytes are expanded during the disease progression. Collectively, this study unravels an unexpected complexity among human circulating monocytes and highlights the existence of specialized populations differently engaged depending on the pathological context.

## Introduction

Monocytes represent an essential component of the innate immune system and play central roles both in homeostasis and in pathological conditions (1, 2). Similarly to other mononuclear phagocytes, such as macrophages, monocytes display a peculiar versatility, whereby they can be engaged in quite opposite functions, including promoting inflammation and leading off its resolution phase. Nonetheless, these incredibly plastic cells are mostly exclusively appreciated as precursors of macrophages, both by refilling the tissue-resident ones and differentiating to recruited macrophages (3). Yet, within the mononuclear phagocyte system, monocytes represent the unique population able to operate as both effector and precursor cells (4). In humans, the current monocyte classification distinguishes three major subsets based on the expression of the lipopolysaccharide receptor CD14 and the FcγRIII/CD16: CD14^++^CD16^−^ (classical), CD14^++^CD16^+^ (intermediate) and CD14^lo^CD16^+^ (non-classical) monocytes, comprising respectively 85-90%, ~5% and ~10% of the total circulating monocyte pool (5, 6). However, given that both CD14 and CD16, as well as the vast majority of monocyte markers, are expressed as a continuum along the three subsets, it is often difficult to clearly separate one population from the other. Different studies suggest the addition of further cell markers to the conventional panel to improve subsets definition and purity (7–9). Each subset exerts distinct activities and retains peculiar properties. Classical monocytes present a more proinflammatory phenotype and are involved in immune responses, being capable of efficient phagocytosis, production of reactive oxygen species, response to fungi and bacteria, and secretion of proinflammatory cytokines. Intermediate monocytes are poorly functionally described, but they are characterized by high levels of HLA-DR molecules. Finally, non-classical monocytes primarily remain in the vasculature, where they exert the specific role of patrolling and act as caretakers and sentinels of the vascular tissue (10–15).

Even though the distinction into classical, intermediate and non-classical monocytes stands as a reproducible classification of circulating monocytes, such distinction is now emerging as being too simplistic because it masks the extensive inter-cellular heterogeneity within the three subsets. Recently, single-cell profiling techniques allowing high-resolution comprehension of circulating mononuclear cells at both transcriptomic and proteomic levels have evidenced a broader complexity and have begun dissecting monocyte diversity (16–22). However, an exact task-division among the different subsets has not been defined yet.

In view of the remarkable lack of consensus on monocyte emerging populations and the growing evidence on their relevance in physiological and pathological contexts, in this study we addressed to deepen our understanding of the variety of human circulating monocytes by single-cell RNA sequencing, which do not require *a priori* knowledge of the markers tested for cell classification and analysis. Therefore, we first investigated circulating monocyte subsets identity and functions in homeostatic conditions. Next, to corroborate the relevance of our findings and determine if alterations in monocyte profile occur during pathological conditions, we investigated circulating monocytes in two distinct pathological contexts, i.e. gastrointestinal cancers and viral infection from severe acute respiratory syndrome-coronavirus-2 (SARS-CoV-2), by using a machine learning model for cluster scRNA-seq data. In both contexts, we clearly recognized all the monocyte subsets previously characterized in homeostasis, but we found disease-specific alterations in terms of frequency and phenotype.

This work provides a single-cell atlas of human circulating monocytes and provides a framework for future studies on the involvement of specific monocyte subsets in health and disease.

## Materials and methods

### Sample collection and cell isolation

Peripheral blood was obtained from 5 adult male healthy donors recruited to the IRCCS-Humanitas Research Hospital. The study protocol was approved by the ethical committee of Humanitas Clinical and Research Center (Prot. Nr 520/18, approved on 9/2018). All participants gave written informed consent. Samples were collected in EDTA-coated tubes (BD Vacutainer K2E), and peripheral blood mononuclear cells (PBMCs) were isolated by Lympholyte^®^ cell separation density gradient solution (Cederlane). Any residual erythrocytes were removed *via* ammonium-chloride-potassium (ACK) Lysing Buffer (Lonza) treatment for 60 sec at room temperature (RT).

### Fluorescence-activated cell sorting (FACS) and single cell sequencing

To assess PBMCs vitality, cells were stained with viability dye (Zombie NIR; Biolegend) for 15 min at RT and Fc-block was performed with 1% human serum for 10 min at RT. PBMCs were then stained for 15 min at RT with fluorophore-conjugated antibodies listed in **Table S1**. Finally, cells were washed in 2% fetal bovine serum/PBS and live CD45^pos^/HLA-DR^pos^/lineage^neg^ (CD3, CD19, CD56) were immediately FACS sorted on a FACSAria III (BD Biosciences) (**Figure S1A**). Cells resuspended in 0.5 ml PBS 1X plus 0.04% BSA were washed once by centrifugation and counted with an automatic cell counter (ThermoFisher; Countess II). About 20,000 cells per sample were loaded into one channel of the Chromium Chip B using the Chromium Single Cell 3’ Reagent Kits (v3 Chemistry) (10X Genomics). 50 ng per sample of the barcoded and amplified cDNA was used then for constructing the sequencing libraries. Sequencing was performed on the NextSeq550 Illumina Platform, generating on average about 477 million reads per sample (on average about 67,000 reads per single cell recovered) following the configuration of the sequencing RUN indicated by the Single Cell 3’ scRNAseq v3 protocol.

### scRNA-seq data processing

FASTQ files were generated by demultiplexing raw base call (BCL) files (mkfastq function, Cell Ranger v.3.0.2) (23). Count function allowed alignment, preliminary filtering, barcode counting, and UMI counting; GRCh38 – hg 38 was used as reference genome.

### Data analysis

Preliminary data filtering, data integration and marker analyses were performed with R (v.3.6.1), using the Seurat package (v.3.1.5) (24). FindCluster function was performed to evaluate clusterings at multiple resolutions (from 0 to 0.5 in steps of 0.05) and results were visualized by using Clustree package (25) (**Figure S1B**). Based on these two algorithms, we used a value of 0.45 for the resolution. In order to annotate clusters, each cell was subjected to the ssGSEA (26, 27) using manually curated signatures. Enrichment pathway analysis was performed with gProfiler (R package) (28) considering only differentially expressed genes (logFC > 0.5 and p-value adjusted < 0.05). Cellular trajectory analysis and activity scores of transcription factors (TFs) were performed with Python using STREAM package (29) and pySCENIC (30), respectively.

### Public gene expression data analysis

Counts matrix of the public scRNAseq dataset from Griffiths et al. (31) and Zhang et al. (32) were obtained from the Gene Expression Omnibus database. Preliminary filtering, data integration and marker analyses were performed as for our dataset (considering 1,000 as variable genes). Monocytes classification was obtained using a machine learning model developed with the caret R package (33), considering a polynomial Kernel support vector machine.

### Statistics

Statistical computations were performed using the software R (v.3.6.1) and the software GraphPad Prism 6 (GraphPad Software). Significance was assigned at p < 0.05, unless stated otherwise. Specific tests are indicated in the relevant figure legends.

### Kaplan-Meier survival analysis

Patient survival was interrogated with the defined cMo infl_3- and the cMo MPA-signature using the online database KMplot (34, 35) survival of a combined cohort of the GSE14210, GSE15459, GSE22377, GSE29272 and GSE51105 datasets. Only patients presenting the selected parameters were considered for the analysis. Gene signatures were designed using *CD14* and *VCAN* as markers of classical monocytes, combined with the top 5 marker genes defined from the HD dataset of either the cMo infl_3 or the cMo MPA cluster.

Further information on methods is reported in the **Supplemental Methods** section.

## Results

### Circulating monocytes are constituted by 9 distinct subsets

To overcome the lack of consensus on human monocyte identity and interrelationship between populations, we performed a scRNA-seq analysis on circulating mononuclear cells from five healthy donors. PBMCs were isolated by density gradient and CD45^pos^/HLA-DR^pos^/lineage^neg^ (CD3, CD19, CD56) cells were FACS sorted, regardless of their expression levels of CD14 and CD16 to avoid the loss of unknown monocyte subsets (**Figure S1A**). The transcriptomes of 35,635 individual cells were analyzed and, after quality control and filtering, a total of 26,474 cells were retained for the analyses, with a median of 65,897 reads and 2,270 genes detected per cell (**Table S2**). Biological replicates showed a significant reproducibility and cell clustering was donor-independent (**Figures S1C, D**).

**Figure 1:**
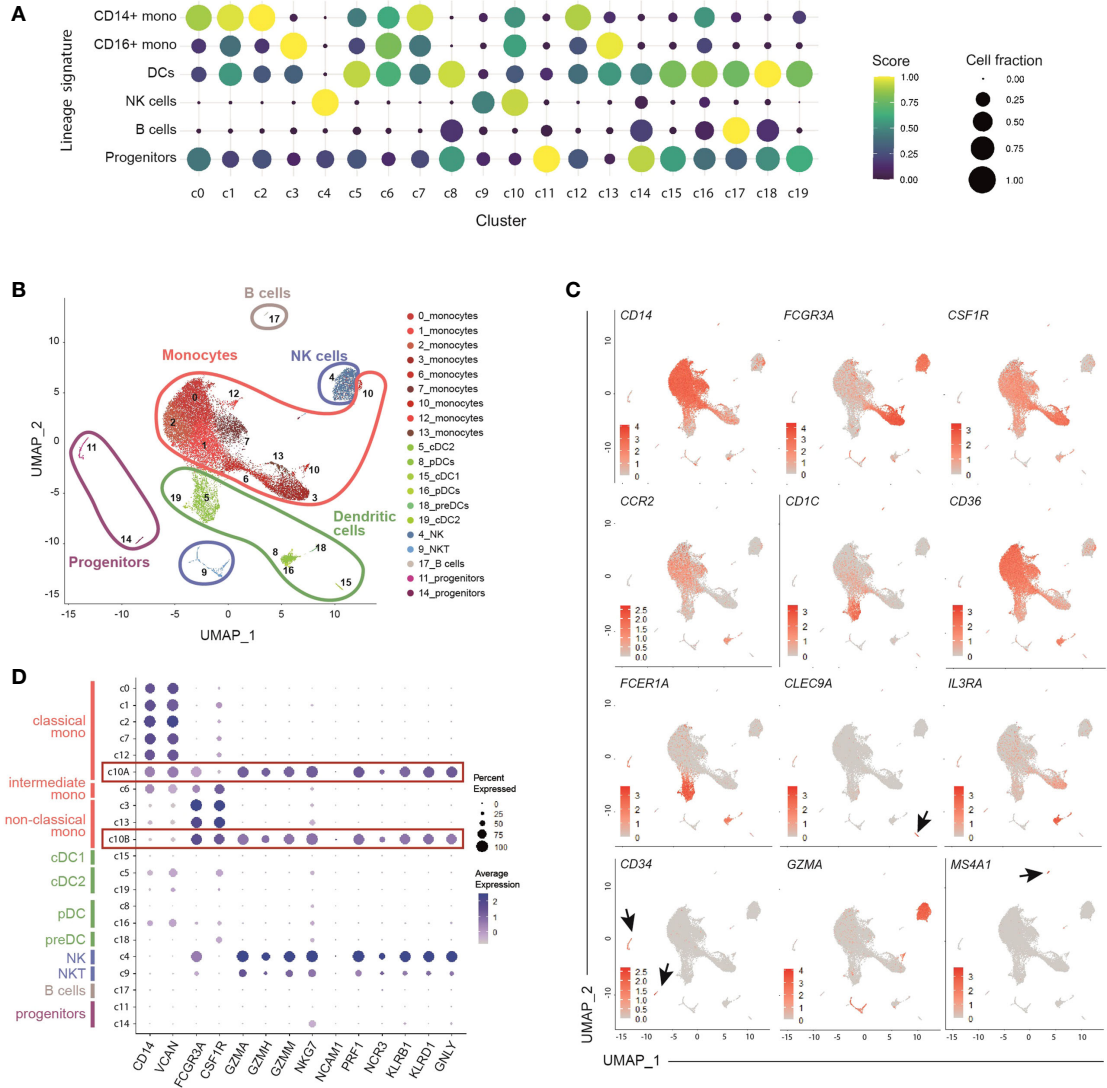
Identification of 9 clusters of circulating monocytes by single-cell RNA sequencing of healthy peripheral blood HLA-DR+ cells. **(A)** Dot-plot showing annotated immune cells by lineage signatures (genes belonging to each signature are listed in **Table S3**). ssGSEA score-based signature expression is colored-coded from blue (lower) to yellow (higher); circle size indicates the fraction of cells expressing the signature. CD14^+^ mono, classical CD14^+^ monocytes; CD16^+^ mono, non-classical CD16^+^ monocytes; DCs, dendritic cells; NK cells, natural killer cells. **(B)** UMAP projection of CD45^+^Lin^-^HLA-DR^+^ cells (n = 26,474) showing 20 clusters belonging to 5 major immune cell subsets. Clusters are numbered according to their size, from the largest (cluster 0) to the smallest (cluster 19). Each dot represents an individual cell. **(C)** Feature plots showing the expression of key genes adopted for manual annotation of myeloid cell clusters. Gene expression is colored-coded from gray (lower) to red (higher). **(D)** Dot-plot showing the expression of key genes adopted for manual annotation of clusters 10A and 10B. Gene expression is colored coded from gray (lower) to blue (higher); circle size indicates the fraction of cells expressing the gene.

Unsupervised clustering identified 20 distinct populations that were annotated according to score enrichment of canonical gene signatures (**Table S3**). Monocytes, dendritic cells (DCs), NK cells and progenitor cells were present. 6 clusters of dendritic cells, including both conventional (cDCs) and plasmacytoid DCs (pDCs), were identified (**Figures 1A, B**). Type 1 cDCs expressed high levels of *HLA*, *CLEC9A*, and *CADM1* (c15), while type 2 cDCs had high levels of *CD1C*, *FCER1A*, and *CLEC10A* (c5, c19) (36) (**Figures 1C** and **S1E**). Based on the expression of *IL3RA/CD123*, *CLEC4C* and *NRP1*, 2 clusters (c8, c16) were recognized as pDCs (36) (**Figures 1C** and **S1E**). We also detected a small cluster (c18) of *AXL*-expressing pre-DCs (16), with a profile similar to pDCs (**Figure S1E**). Two small clusters (c11 and c14) showed a distinct set of genes including *CD34*, *GATA1*, *GATA2*, and *SOX4*, which allowed their annotation as common myeloid progenitors (**Figures 1C** and **S1E**). Finally, 2 clusters of NK cells (c4, c9) and 1 of B cells (c17) were identified (**Figures 1A, B**).

Among monocytes, we identified 9 distinct clusters, indicating that the current classification only partially captures the heterogeneity of circulating monocytes. Five clusters (c0, c1, c2, c7, c12) included classical monocytes, based on the expression of *CD14*, *VCAN*, *NCF1* and *MS4A6A*; 2 clusters (c3, c13) were characterized by high levels of *FCGR3A/*CD16, *LST1*, *RPS19*, *MS4A7*, and *CSF1R*, and were identified as non-classical monocytes (37–40); 1 cluster (c6) was annotated as intermediate monocytes given their mid-levels of the above-mentioned genes compared to the classical and non-classical subpopulations (**Figures 1A, C, D** and **S1E, F**). Monocytes belonging to c10 located in proximity to NK cells in the Uniform Manifold Approximation and Projection (UMAP) analysis (**Figure 1B**) and, similarly to NK cells, exhibited a cytotoxic signature (*GZM* genes and *PRF1*) (**Figure 1D**). Using graph-based clustering (**Figure S1G**), c10 resulted constituted of both classical (c10A) and non-classical (c10B) monocytes (**Figure S1H**).

### Phenotypic characterization of newly identified monocyte subsets

**Figure 2:**
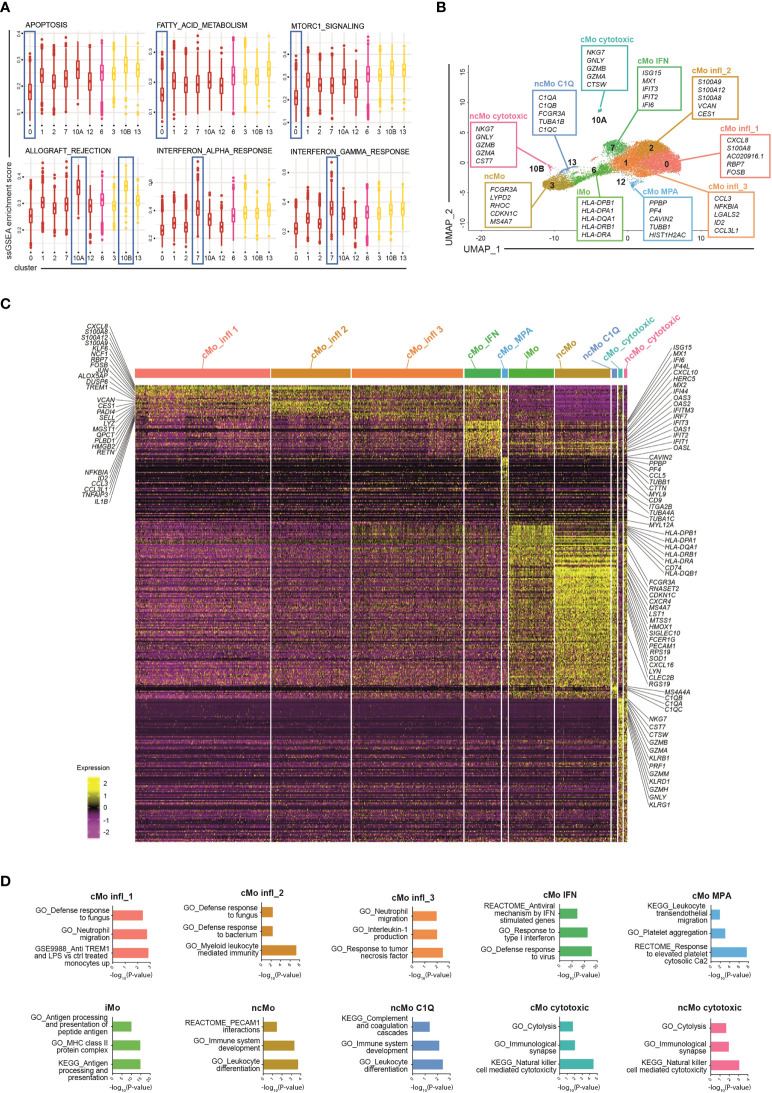
Phenotypic characterization of human blood monocytes in steady state conditions. **(A)** Boxplot showing the normalized enrichment score (ssGSEA) of selected Hallmark signatures found differentially enriched in clusters belonging to the same monocyte macro-group (classical and non-classical monocytes). Colour code: dark red, classical monocytes; pink, intermediate monocytes; yellow, non-classical monocytes. **(B)** UMAP projection of monocyte clusters (c0, c1, c2, c3, c6, c7, c10A, c10B, c12 and c13). Up to five cluster marker genes are listed in boxes next to each cluster. Cluster marker genes are defined as in Material and Methods. **(C)** Single cell gene expression heatmap showing significant differentially expressing genes (*pvalue_adj_* ≤ 0.05, *pct* 1 ≥ 0.1, *pct*2 ≥ 0.1, *log*_2_ *FC* > 0.5) among monocyte cell subsets. Selected gene names are labeled; gene expression is colored-coded from purple (lower) to yellow (higher); gene expression level is scaled by row. **(D)** Pathway analysis of differentially expressed genes (one cluster of monocyte vs all the other monocyte cells). Bar plots show key canonical pathways collected in The Molecular Signatures Database (MSigDB) enriched in individual populations of monocytes. The Canonical Pathways gene sets derived from the KEGG pathway database, the Canonical Pathways gene sets derived from the Reactome pathway database and the Gene Ontology gene sets were taken into consideration. Only upregulated genes were considered.

To dissect monocyte heterogeneity, we then applied the single-sample Gene Set Enrichment Analyses (ssGSEA) (26, 27) of the Hallmark gene sets (41) (**Figures 2A** and **S2A**). Clusters belonging to either the classical or the non-classical subsets shared common functional properties, supporting the current monocyte classification. Clusters of classical monocytes were enriched for transcripts related to angiogenesis, epithelial-mesenchymal transition and wound healing pathways, while the Notch signaling pathway was enriched in non-classical monocytes (**Figure S2A**). Other functional pathways, however, were selectively enriched in distinct clusters regardless of the specific macro-category they belong to. In particular, cells in the most abundant cluster (c0, comprising 27.7% +/- 6.6% of all monocytes) showed lower activation of apoptosis, fatty-acid metabolism, and mTORC1 signaling pathways as compared to other classical monocytes (**Figure 2A**). This cluster, as well as c1 and c2 (comprising 23% +/- 2.5% and 16.2% +/- 3.6% of all monocytes, respectively), displayed a proinflammatory phenotype. In particular, c0 and c2 were strictly related (**Figure S2B**) and expressed the highest levels of proinflammatory genes, such as *S100A8/A9/A12* (**Figure S2C**) and were therefore operationally defined them as cMo infl_ (classical monocyte, inflammatory, subset_) 1 and 2, respectively. Also c1 (cMo infl_3) exhibited a functional activation phenotype and proinflammatory features. Indeed, among its top cluster marker genes, we identified inflammatory cytokines (*CCL3*, *CCL3L1*, and *IL1B*) and *NFKBIA*, a positive regulator of NF-kB activity (42) (**Figure 2B**). In the analysis of differentially expressed genes (DEGs), neutrophil migration pathway was increased in both cMo infl_1 and cMo infl_3, consistent with the upregulation of *CXCL8* and *CCL3* in the two clusters, respectively (**Figures 2C, D**). Cells belonging to cMo infl_2 expressed high levels of genes involved in the antimicrobial defense (*RETN*, *PADI4*, *LYZ*; **Figure 2C**) and showed a significant enrichment of cell defense against bacteria pathways (**Figure 2D**).

The two remaining clusters of classical monocytes displayed peculiar transcriptional profiles as well, highlighting specific functional properties. Cells constituting c7 (7.3% +/- 2.6% of all monocytes, from now cMo IFN) were enriched in genes associated with interferon signaling pathways (*ISG15*, *MX1*, *IFIT2/3*, *IFI6*, *CXCL10*, *HERC5*) and genes belonging to the IFIT, IFI and OAS families (**Figures 2B, C**). Accordingly, signature analyses showed enrichment of pathways involved in IFNα/γ responses and defense against viruses (**Figure 2D**). The profile of c12 (1.2% +/- 0.6% of all monocytes) was instead associated with leukocyte transendothelial migration genes (*MYL9*, *MYL12A*, *CD9)* (**Figures 2C, D**) and platelet-related genes (*PPBP* and *PF4*) (**Figure 2B**) and showed significant upregulation of platelet-associated pathways (**Figure 2D**), suggesting that this cluster (from now cMo MPA) may correspond either to circulating monocyte-platelet aggregates (43, 44) or to the recently reported megakaryocyte-like monocyte subset (45).

Most of the non-classical monocytes grouped into c3 (from now ncMo) and a minor fraction clustered independently in c13 (12.2% +/- 5.5% and 1.2% +/- 0.6% of all monocytes, respectively). They expressed a very similar transcriptome, characterized by high levels of cell activation (*LST1*, *LYN*, *SIGLEC10*, *SOD1*, *RPS19*) and cell cycle regulation genes (*MTSS1*, *CDKN1C*) (**Figure 2C**). c13 (from now ncMo C1Q) was characterized by the highest expression of the complement genes *C1QA*, *C1QB* and *C1QC* and their related pathways (**Figures 2C, D** and **S2D**), consistent with their involvement in complement-mediated phagocytosis (12, 38).

Intermediate monocytes were all included in c6 (from now iMo), comprising 9.4% +/- 1.5% of the total monocytes and were strongly characterized by high levels of HLA genes in MHC class II (12) and enrichment of antigen presentation pathways (**Figures 2C, D**). Finally, cells included in c10A-B (0.9% +/- 0.5% and 0.8% +/- 1% of all monocytes, respectively) presented upregulation of cytolysis and cell death pathways (**Figure 2D**), and likely corresponded to the recently identified NK-like monocyte subset with cytotoxic activity (45).

### Monocyte subsets developmental relationship

**Figure 3:**
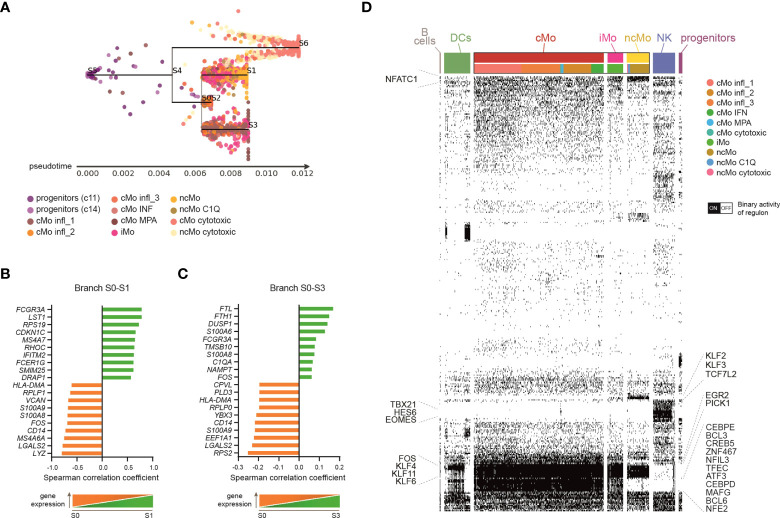
Monocyte subsets developmental relationship. **(A)** Subway map plot visualization of inferred developmental trajectory of myeloid progenitors and monocytes by STREAM. Cells are colored according to the cluster of origin as shown in the legend. Starting from myeloid progenitors, cells bifurcated into Mo cytotoxic (branch S4-S6) and a branch that further separated into three sub-branches: classical monocytes (belonging to c1, c2, c7, c12) primarily localize into node S0 and branch S0-S2; branch S0-S3 is mainly consisted of classical monocytes from c0; branch S0-S1 identify a clear directional progress from the intermediate to the non-classical monocytic cells. **(B, C)** Bar plots showing the top-20 transition markers significantly most correlated with the pseudotime calculated in branch S0-S3 **(B)**, containing classical monocytes from c0, or branch S0-S1 **(C)**, containing intermediate and non-classical monocytes from c6, c3 and c13. 10 genes positively and 10 genes negatively correlated are shown. **(D)** Single cell binary matrix showing the activity of each regulon in each cell (black blocks represent cells that are “on”, white blocks cells that are “off”). TFs belonging to selected regulons active in monocytes are labeled.

Many studies have demonstrated that classical monocytes are able to give rise to the non-classical population in mice (46–48). Recently, the same program has been shown also in humans (6), even though the hypothesis that some CD14^+^CD16^-^ cells can arise following another route of development cannot be excluded (1). Since we found several classical and non-classical subsets of monocytes under homeostasis, we asked whether their developmental paths were connected and how. To this aim, a Single-cell Trajectories Reconstruction, Exploration And Mapping (STREAM) analysis (29) was performed, taking advantage of the presence of precursor cells within the dataset. The sequential progression of classical, intermediate and non-classical monocytes along the pseudotime trajectory, as well as the transition markers of branch S0-S1, were in line with the hypothesis of a sequential transition from CD14^+^CD16^−^ *via* CD14^+^CD16^+^ to CD14^lo^CD16^+^ monocytes (**Figures 3A, B** and **S3A, B**). Interestingly, there was a clear separation between the branch S0-S3, which was mainly composed of cells from cMo infl_1, and the shorter branch S0-S2, constituted by the other classical monocytes (**Figures 3A** and **S3A**), suggesting that cMo infl_1 was less engaged in biological processes compared to the other subsets and possibly maturing toward a specific proinflammatory phenotype. This is in line with the upregulation of the ferritin heavy/light chains (*FTH1/FTL*), the mediator of inflammation nicotinamide phosphoribosyltransferase *NAPMT* (49), and the calcium binding proteins *S100A6/A8* (**Figure 3C**). Notably, c10A and c10B (from now cMo cytotoxic and ncMo cytotoxic, respectively) separated from the other monocytes early along the trajectory pseudotime (**Figures 3A** and **S3A**).

Single-cell regulatory network inference and clustering (SCENIC) (30) allowed us to investigate the involvement of specific TFs in driving monocyte transcriptomic variability. Besides common monocytic transcription factors (TFs), such as SPI1 (PU.1) and Kruppel-like factor 4 (KLF4) (50, 51), some TFs resulted selectively activated in classical or non-classical monocytes (**Figure 3D**), suggesting that the transition from CD14^+^ to CD16^+^ cells requires the activity of specific regulons. For example, classical monocytes displayed active CCAAT/enhancer binding proteins (CEBPD/E), BCL3/6 and Nuclear Factor, Interleukin 3 Regulated (NFIR3). In contrast, KLF2/F3 and TCF7L2 were highly activated in non-classical monocytes. Of note, TFs known for their role in NK cell biology, such as TBX21 (T-bet), HES6 and EOMES (52), were selectively activated in c10, suggesting that these TFs support the profile of cytotoxic monocytes.

### Relevance of distinct monocyte subsets in cancer patients and in response to immunotherapy

**Figure 4:**
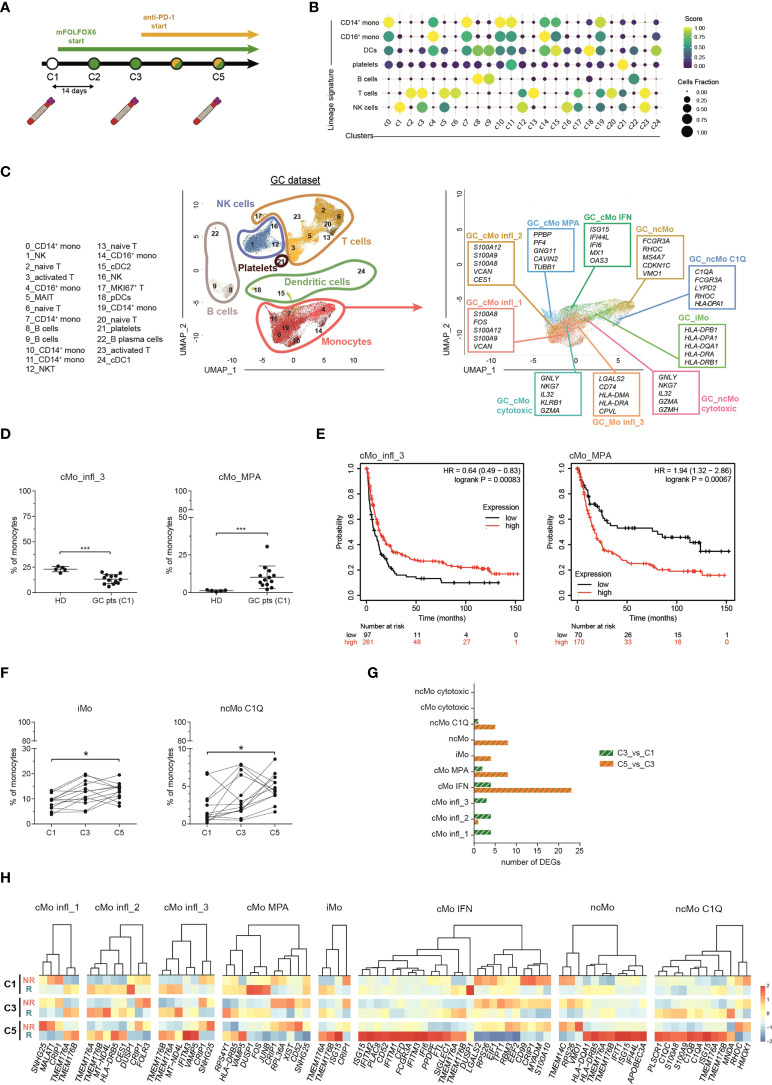
Monocyte subsets in advance gastrointestinal cancers and response to immunotherapy. **(A)** Schematic illustration of the clinical trial treatment strategy adopted in the study of Griffiths and colleagues (Mod. from Griffiths et al., PNAS, 2020). Advanced GC pts received mFOLFOX6 chemotherapy at the beginning of the trial for 2 cycles (14 days per cycle). From cycle 3 through 12, they received the combination of mFOLFOX6 and anti-PD-1 immunotherapy. Blood samples were collected at C1 (cycle 1, baseline), C3 (cycle 3) and C5 (cycle 5). PBMCs were isolated and frozen. Single cell RNA sequencing was performed on the cryopreserved PBMCs samples using 10X Genomics technology and sequenced on an Illumina HiSEq. **(B)** Dot-plot showing annotated immune cells by lineage signatures (genes belonging to each signature are listed in **Table S4**). ssGSEA score-based signature expression is colored-coded from blue (lower) to yellow (higher); circle size indicates the fraction of cells expressing the signature. (**C**, left) UMAP projection of GC pts PBMCs (n = 55,293) showing 25 clusters individually annotated belonging to 6 major immune cell subsets. Clusters are numbered according to their size, from the largest (cluster 0) to the smallest (cluster 24). Each dot represents an individual cell. (**C**, right) UMAP projection of GC pts monocytes clustered using the machine learning classifier. Up to five cluster marker genes are listed in boxes next to each cluster. Cluster marker genes are defined as in Material and Methods. **(D)** Percentage of cMo_infl_3 and cMo_MPA over the total monocyte population from healthy donors and GC pts at C1. Statistical significance was determined by the Mann-Whitney test. (***) P < 0.001. **(E)** Kaplan-Meier curves of patient first progression survival defined by the cMo infl_3 signature (left) or the cMo MPA signature (right) using KMplot (34). Significance was evaluated by the log-rank Mantel–Cox test. **(F)** Percentage of iMo and nc_Mo C1Q over the total monocyte population in GC pts during therapy. Each line represents a patient percentage trend along the three therapy cycle steps. Statistical significance was determined by the Friedman test followed by Dunnett’s multiple comparison test. (*) *P* < 0.05. **(G)** Bar plot showing the number of significant differentially expressing genes after immunotherapy (green and white bars) and immunotherapy (orange and green pathways) in monocyte subtypes. **(H)** Heatmap showing the gene expression in C1, C3 and C5 of significant differentially expressing genes between responders and non-responders after immunotherapy. Gene expression is colored-coded from blue (lower) to red (higher); gene expression level is scaled by columns. C1, cycle 1 = baseline; C3, cycle 3 = chemotherapy mFOLOFX6 regimen; C5, cycle 5 = chemotherapy + anti–PD-1 immunotherapy. HD, healthy donors; GC pts, gastrointestinal cancer patients. NS, non-responder patients; R, responder patients.

The frequency of peripheral blood cells represents a non-invasive indicator of immunotherapy responsiveness in cancer patients (53). To investigate the dynamics of monocyte subsets in response to checkpoint inhibitors, we inspected a published scRNA-seq dataset of cancer patients from Griffiths et al. (31), hereinafter referred as “GC dataset”). This dataset contains transcriptomes of PBMCs collected from 13 advanced (stage 3/4) gastrointestinal cancer patients (phase I clinical trial, NCT02268825) at 3 time points: before treatment (C1), after two cycles of chemotherapy (mFOLFOX6) (C3) and after two cycles of mFOLFOX6 and anti-PD-1 antibody (C5) (**Figure 4A**). We applied the same strategy used in our scRNA-seq of healthy donors (hereinafter referred as “HD dataset”) to the GC dataset and obtained 55,293 cells, comprising B cells, T cells, NK cells, dendritic cells, monocytes and platelets (**Figures 4B, C** and **S4A**). We then considered only monocytes and, splitting the HD dataset into a training set (80% of cells) and a test set (remaining 20% of cells), we developed a machine learning model for clustering analysis (**Figure S4B**). With this approach, we could clearly recognize in the GC dataset all the 9 monocyte clusters previously identified in homeostatic conditions, confirming the robustness of the machine learning approach and indicating that monocyte subpopulations are broadly conserved in steady-state and in gastrointestinal cancer patients (**Figure 4C**). Compared to healthy donors, cancer patients showed a similar frequency of most monocyte subpopulations (**Figure S4C**), with the exception of cMo infl_3 and cMo MPA which were found decreased and increased, respectively (**Figure 4D**). To explore the relevance of these monocytes in cancer patients, we derived two gene signatures representative of each cluster and tested them as predictive of first progression survival, in a database of 358 patients. Of note, patients with above median level expression of the cMo infl_3-signature had an improved survival (logrank P=0.00083; n=354), while above median level expression of the cMo MPA-signature correlated with worst first progression survival (logrank P=0.00067; n=240), highlighting the influence of such monocyte subsets on patient outcome (**Figure 4E**). We then investigated the impact of monocytes on the response to treatments and found that anti–PD-1 therapy selectively increased the relative frequency of the ncMo C1Q and iMo subsets (**Figures 4F** and **S4D**) and the expression of the complement genes *C1QA/B/C* exclusively in ncMo_C1Q from responders (**Figure 4H**).

In their work, Griffiths et al. found higher activation of growth factors, inflammation, and differentiation pathways in monocytes from responders *versus* non-responders before treatment (31), with the following trend: reduction of the score after immunotherapy, further reduction in patients responsive to treatment and a significant enrichment in non-responders after anti-PD-1 immunotherapy. When the same pathways were examined in each cluster individually (**Figure S4E**), they were not equally enriched or follow the same trend in all the monocyte subpopulations. cMo IFN was the most altered cluster after immunotherapy (**Figure 4G**), with interferon-stimulated genes (*IFITM1/2/3* and *ISG15*) significantly overexpressed in responder patients compared to non-responders (**Figure 4H**). Increased expression of IFN-related genes (*ISG15*, *IFI44L* and *IFI6*) was also evident in intermediate and non-classical monocytes from responders (**Figure 4H**), in line with their upregulation of IFN pathways in homeostatic conditions (**Figure 2A**). Conversely, other clusters did not show significant alterations among different time points nor between the two groups of patients. Overall, these data confirm that distinct circulating monocyte subsets are differentially engaged in cancer patients and can play different roles in their response to immunotherapy.

While investigating the prognostic potential of cMo IFN in cancer patients, we observed significant overexpression of *TMEM176A* and *TMEM176B* genes in cMo IFN from responders, though both genes were differentially expressed even before treatment (**Figure S5A**). *TMEM176A/B* are co-regulated genes encoding transmembrane proteins belonging to the MS4A family. They have been detected in monocytes and macrophages (54), dendritic cells and RORγt^+^ lymphocytes (55). In our study we found the highest levels of these genes in circulating monocytes, with different expression depending on the subpopulations, while lower levels in dendritic cells (**Figure S5B**), suggesting a previously unappreciated role in monocytes and candidating TMEM176A/B as biomarkers of response to immune-checkpoint inhibitors.

### Activated cytotoxic monocytes are expanded in COVID-19 patients

**Figure 5:**
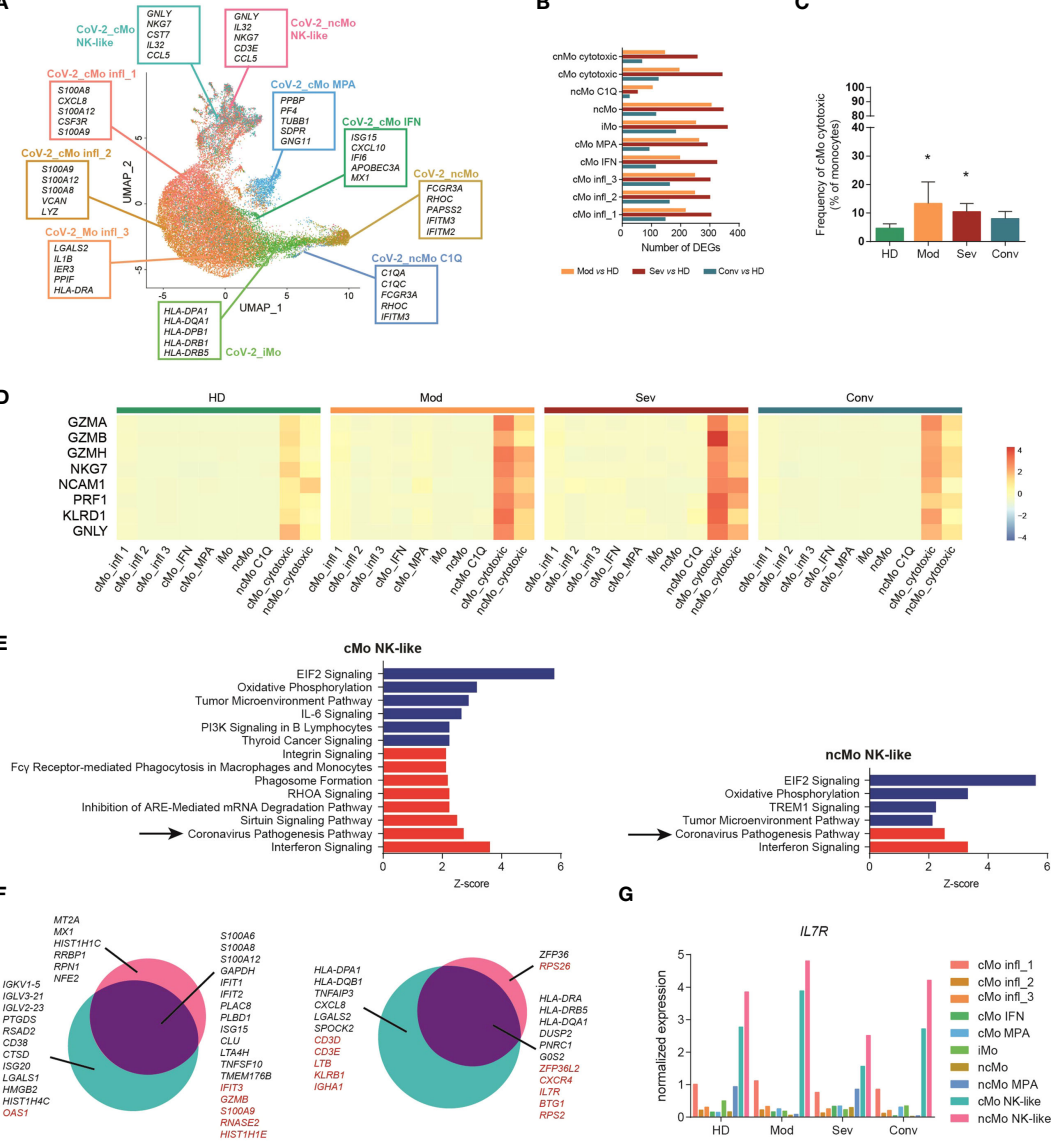
Cytotoxic monocytes are involved in COVID-19. **(A)** UMAP projection of Cov-2 pts monocytes clustered using the machine learning classifier. Up to five cluster marker genes are listed in boxes next to each cluster. Cluster marker genes are defined as in Material and Methods. **(B)** Bar plot showing the number of DEGs between monocytes from COVID-19 patients with different pathological conditions and healthy donors. **(C)** Percentage of cMo cytotoxic over the total monocyte population from healthy donors and Cov-2 pts. Statistical significance was determined by the Dunn’s multiple comparisons test. (*) P < 0.05. **(D)** Heatmap showing the expression of selected cytotoxic genes in healthy donors (HD), patients with severe (sev) or moderate (mod) COVID-19 and convalescent patients with COVID-19 (conv). Gene expression is colored-coded from blue (lower) to red (higher); gene expression level is scaled by row. **(E)** Ingenuity pathway analysis of the DEGs between cytotoxic monocytes from severe COVID-19 pts and healthy donors. Only pathways that were significantly (-log10(p-val) > 1.3) upregulated (z score > 2, in red) or downregulated (z score < -2, in blue) are shown. Analysis of the other monocyte clusters are shown in **Figure S7** **(F)** Venn diagram showing the distribution of DEGs in cytotoxic monocytes between moderate and severe patients. Genes in black are differentially expressed also in other monocyte subpopulations; genes in red are differentially expressed exclusively in cytotoxic monocytes. **(G)** Bar plot showing the expression of IL7R in each cell cluster of the CoV-2 dataset. HD, healthy donors; sev, severe COVID-19 patients; mod, moderate COVID-19 patients; conv, convalescent patients.

Monocytes are pivotal players in viral infections, including COVID-19 (56). We investigated the transcriptomic changes of monocyte subpopulations in a public single-cell transcriptomic dataset of PBMCs from COVID-19 patients at different stages of the disease (hereafter named “CoV-2 dataset”) (32) (**Figure S6A**). Monocytes were identified among other mononuclear cells (**Figures S6B-D**) and re-clustered applying the machine learning approach used for the GC dataset, allowing the recognition of all the 9 subpopulations of monocytes (**Figure 5A**). The phenotype of most subtypes was highly altered according to the severity of disease and, in most clusters, severe patients counted the highest number of DEGs when compared to healthy donors, while a progressive lower number of DEGs was detected in moderate and convalescent patients, respectively (**Figure 5B**). Interestingly, all subsets showed comparable frequencies (**Figure S6E**), with the only noticeable exception of cytotoxic monocytes, which were significantly expanded in patients with moderate or severe disease conditions compared to healthy controls (**Figure 5C**). Moreover, perforin and granzymes, as well as other cytotoxic genes, were selectively upregulated in cMo- and ncMo cytotoxic populations according to the severity of disease (**Figure 5D**).

To better investigate the role of circulating monocytes in COVID-19, we performed Ingenuity Pathway Analysis (IPA) [QUIAGEN lnc., https://digitalinsights.qiagen.com/IPA, (57)], and found that, compared to healthy donors, the most significantly downregulated pathways in all the pathological conditions were the Oxidative Phosphorylation signaling, Tumor Microenvironment signaling categories and Translation Initiation Factor 2 (eIF2) signaling, while PD-1, PD-L1 Cancer Immunotherapy Pathway was downregulated in the severe pathological group (**Figure S7**). A significant upregulation of the interferon signature was detected in all monocytes from patients with COVID-19, but surprisingly, in patients with severe disease, the Coronavirus Pathogenesis Pathway was selectively upregulated only in the cMo- and ncMo cytotoxic monocyte subsets (**Figure 5E**). We further examined this pathway and noted that, while both cytotoxic monocyte subsets showed a significant upregulation in the response to interferon type I and activation of IRF7 pathways in severe patients as compared to healthy controls, cMo- and ncMo cytotoxic monocyte subsets showed an opposite regulation of the NALP3 inflammasome pathway, with upregulation of CASP1, encoding the protease essential for the production of the active and mature form of IL-1β (58), only in cMo cytotoxic (**Figure S6F**). We then explored if the phenotype of cytotoxic monocytes differed depending on disease severity. Looking at differentially expressed genes between the cytotoxic subsets from moderate and severe patients, we found many genes modulated in the same way of other clusters, including the upregulation of *TNFAIP3*, *CXCL8* and different HLA genes in moderate patients, and upregulation in severe patients of inflammatory cytokines, such as *S100A6*, *S100A8*, *S100A12*, and the interferon-related genes *IFIT1* and *IFIT2* (**Figure 5F**). Of note, among genes selectively modulated in cytotoxic monocyte subpopulations but not in the others, we identified the IL-7 receptor (*IL-7R*) (**Figure 5F**). Since the recombinant human IL-7 (rhIL-7) has been evaluated as promising vaccine adjuvant against SARS-Cov-2 (59), we asked whether *IL-7R* is expressed by all monocytes and upregulated only in the cytotoxic monocytes from moderate patients or if the receptor is selectively expressed by this subpopulation. Interestingly, while other subsets showed low levels of *IL-7R*, both cMo and ncMo cytotoxic resulted as the most expressing cells (**Figures 5G**).

These results point to cytotoxic monocyte subsets as new players in COVID-19 and suggest they contribute to the disease pathogenesis through both common and distinct effector pathways.

## Discussion

The involvement of monocytes in physiological and pathological conditions has been primarily related to their role as reservoir of macrophages and monocyte-derived DCs, while their role as effector cells has been overlooked. Recent studies have brought to light the heterogeneity of myeloid cells and their plasticity (16, 17, 21, 22, 45, 60, 61), nevertheless circulating monocytes remain poorly characterized, and the classification based on their expression of CD14 and CD16 (5) holds as the most credited one. Here we unraveled the transcriptional landscape of human circulating monocytes in homeostatic conditions and we investigated their relevance in pathological states inspecting public scRNA-seq datasets through a machine learning model for clustering analysis.

Among the classical monocytes, the three most abundant subpopulations displayed all inflammatory programs and activation states, and were closely related. Interestingly, cMo infl_1 and cMo infl_2 had a profile similar to neutrophil-like monocytes (NeuMo) (62) and, by trajectory analysis, emerged as two clusters captured at different states of maturation. The developmental paths of monocyte populations also confirmed the sequential transition from classical, intermediate and non-classical subset (6), although cytotoxic monocytes followed peculiar development and differentiation routes. Specific TFs, selectively active in either classical (CEBPD, CEBPE, BCL3/6, NFIR3), non-classical (KLF2, KLF3, TCF7L2) or cytotoxic (TBX21, HES6, EOMES) monocytes, are possibly involved in the differentiation from one monocyte population to the other.

In cancer patients, specialized monocyte subtypes caught our attention as relevant players during disease progression. Interestingly, the frequency of cMo MPA was significantly increased in cancer patients compared to healthy subjects, possibly as a manifestation of the inflammatory milieu characterizing the tumor condition (63), and enrichment of cMo MPA-signature in cancer samples correlated with significant worst prognosis. On the contrary, cMo infl_3 showed the exact opposite behaviour, suggesting a protective function of these cells in disease. Instead, considering the effect of the anti-PD-1 immunotherapy, we recorded an expansion of iMo [in line with the increased MHC II gene expression observed by Griffiths et al. (31)]. Both cMo IFN and ncMo C1Q were involved in response to therapy. Particularly, cMo IFN, which showed a discriminative type I IFN-induced signature, resulted the major altered subtype after anti-PD-1 treatment, and cells from responder patients showed significant upregulation of interferon genes compared to non-responders. Other studies have investigated the correlation of interferon-related gene signature with clinical response to immune checkpoint blockade therapies (64, 65). Our results based on analyses of circulating monocytes may provide rationale to design non-invasive strategies to predict clinical response to anti-PD-1 therapy.

Sometimes the boundaries between distinct immune cell populations are blurred. This is for instance the case of c10, which we annotated as cytotoxic monocytes because of their similarity with NK cells. Villani et al. had previously described a subset of monocytes with a distinct cytotoxic gene signature (16), but subsequent analyses of the same dataset suggested a misclassification of clusters, probably due to the experimental strategy adopted (66). Cytotoxic monocytes were reported in a more recent study of scRNA-seq, both in homeostatic conditions and in Vogt-Koyanagi-Harada disease (45). In our hands, monocytes with a marked NK-like signature were deeply modulated in COVID-19 patients, showing strong transcriptomic alterations, cellular activation and interferon response depending on disease severity and stage. The Coronavirus Pathogenesis Pathway selectively upregulated in cMo cytotoxic and ncMo cytotoxic from severe COVID-19 patients compared to healthy controls. Accordingly, we also found a disease severity-dependent upregulation of cytotoxic genes specifically in these two subsets. This observation is in line with the significant upregulation of granzyme B and perforin proteins in monocytes from COVID-19 patients compared to healthy donors reported by Ahmadi P. et al. (67). Moreover, despite the fact that the literature reports a reduction of non-classical monocytes in COVID-19 patients (68), in our dataset the only subpopulation altered in frequency was the one of cMo cytotoxic monocytes, which increased in severe and moderate patients. Finally, our data showing the expression of IL-7R in cytotoxic monocytes are consistent with the presence of IL7R^+^ monocytes/macrophages in COVID-19 BALF (69), and further support the hypothesis that these cells are key players in the progress of the disease.

Taken together, our results provide a framework for analyzing circulating monocytes in pathology. Therapeutic interventions designed to target selective monocyte subsets may offer opportunities to enhance treatment efficacy. The significance of each of the monocyte subpopulations will have to be validated in functional studies, and further studies are needed to define the role of distinct monocytes as precursors of specific macrophage subpopulations in tissues.

## Data availability statement

The dataset generated for this study can be found in the GEO database under accession number GSE213004. Further inquiries can be directed to the corresponding author.

## Ethics statement

The studies involving human participants were reviewed and approved by IRCCS Istituto Clinico Humanitas - Humanitas Mirasole S.p.A. The patients/participants provided their written informed consent to participate in this study.

## Author contributions

Conception and Design: ML, FM. Technical support: FC, CP. Methodology: AR, AC, MV, CP, ST. Analysis and interpretation of data: AR, AC. Writing – original draft: ML, FM, AR. Writing – review & editing: AC. Study supervision: ML, FM. All authors contributed to the article and approved the submitted version.

## Funding

The research leading to these results has received funding from AIRC (Associazione Italiana per la Ricerca sul Cancro) under IG 2020 - ID. 24393 project - P.I. F. Marchesi, and under fellowship - ID 25290 – 2020 – to AR; from Italian Ministry of University and Research (PRIN 2017 - 20174T7NXL – ML) and Ministry of Health (Ricerca Finalizzata COVID-2020-12371849 – P.I. ML).

## Acknowledgments

We thank the Humanitas Flow Cytometry Core and the Humanitas Genomic Unit and Sequencing Facility, in particular Dr. Javier Cibella. We also thank Dr. Domenico Mavilio for his critically reading of the manuscript and for his insightful comments.

## Conflict of interest

The authors declare that the research was conducted in the absence of any commercial or financial relationships that could be construed as a potential conflict of interest.

## Publisher’s note

All claims expressed in this article are solely those of the authors and do not necessarily represent those of their affiliated organizations, or those of the publisher, the editors and the reviewers. Any product that may be evaluated in this article, or claim that may be made by its manufacturer, is not guaranteed or endorsed by the publisher.

## Abbreviations

C1Q, complement component 1, q subcomponent; cDC, conventional dendritic cell; cMo, classical monocyte; COVID-19, Coronavirus disease 2019; DC, dendritic cell; DEGs, differentially expressed genes; FACS, fluorescence-activated cell sorting; GC, gastric cancer; HD, healthy donor; HLA, human leukocyte antigen; IFN, interferon; iMo, intermediate monocyte; infl_1, inflammatory, subset 1; infl_2, inflammatory, subset 2; infl_3, inflammatory, subset 3; IPA, Ingenuity Pathway Analysis; MHCII, major histocompatibility complex, class II; MPA, monocyte-platelet aggregates; ncMo, non-classical monocyte; NeuMo, neutrophil-like monocytes; NK cells, natural killer cells; PBMCs, peripheral blood mononuclear cells; pDC, plasmacytoid dendritic cell; SARS-CoV-2, severe acute respiratory syndrome coronavirus 2; SCENIC, Single-Cell rEgulatory Network Inference and Clustering; scRNA-seq, single-cell RNA sequencing; ssGSEA, single sample Gene Set Enrichment Analysis; STREAM, Single-cell Trajectories Reconstruction, Exploration And Mapping; TF, transcription factor; UMAP, Uniform Manifold Approximation and Projection.
