# Supplementary material for: Distinct responses of newly identified monocyte subsets to advanced gastrointestinal cancer and COVID-19
Source: Front Immunol. 2022 Oct 3;13:967737. doi: 10.3389/fimmu.2022.967737 (PMC9576306; doi:10.3389/fimmu.2022.967737)
Supplement: Supplementary file 1 [file DataSheet_1.docx]

Supplementary Material

SUPPLEMENTAL METHODS

## Data analysis (HD dataset)

***Filtering***

Only genes expressed by at least 3 cells and only cells expressing at least 500 genes were taken into consideration. Moreover, cells were filtered considering mitochondrial expression genes (cells with a mitochondrial expression lower than 10% were considered).

***Sample integration and scaling***

In order to integrate the 5 samples, for each one 2,000 variable genes were determined (FindVariableFeatures; vst method) and used as anchors in FindIntegrationAnchors function. Data were log normalized and scaled (scaling factor: 10,000). A matrix of 21,507 genes per 26,474 cells was obtained.

***Clustering***

Clusters were set out with the Louvain Algorithm (FindClusters) function with a resolution of 0.45. The average expression matrices were calculated through the AverageExpression() function. Only cells expressing genes participate in mean calculation.

***Clusters annotation***

The gene signatures adopted for cluster annotation are listed in **Table S1**.

An enrichment score matrix $\boldsymbol{S}_{\boldsymbol{k,z}}$ was calculated with ssGSEA [1]. When negative values occurred, 0 values were imposed. An arithmetic mean vector $M=[m_{1}, \ldots, m_{k}]$ was defined, in which a generic element $m_{i}, where i \epsilon\left[ 1,k \right],$ was obtained as $m_{i}=mean(\boldsymbol{S}_{i,1:z})$. When $\boldsymbol{S}_{i,j}<m_{i}$ we imposed $S_{i,j}=0$, considering$j \epsilon\left[ 1,z \right]$. Let $r$ be the number of clusters (20 in this paper) and $w \epsilon\left[ 1,r \right]$*,* we defined:

- The cell fraction matrix $\boldsymbol{C}\boldsymbol{F}_{\boldsymbol{k,r}}$, $CF_{i,w}=\frac{\# cells expressing \Gamma_{i}}{\# cells in the cluster_{w}}$
- The expression score matrix $\boldsymbol{E}\boldsymbol{S}_{\boldsymbol{k,r}}$,$ES_{i,w}=mean(S_{i,cells of cluster_{w}})$.

***Cluster Marker Gene Definition***

We identified cluster marker genes specific to each cluster using the FindAllMarkers() Seurat function, choosing wilcox as the statistical test (**Table S6**). Only genes with $\ln FC$ greater than 0.25 were considered, expressed by at least 3 cells per group (first group: cluster under analysis; second group: all the remaining clusters together). We defined cluster markers only genes with: $pvalue_{adj}\leq0.05, pct1\geq0.1, pct2\geq0.1, \log_{2} FC\geq0.5$.

***single-sample Gene Set Enrichment Analysis (ssGSEA)***

ssGSEA was performed with the packages AnnotationDbi v.1.48.0, org.Hs.eg.db v.3.10.0, qusage [2], and GSVA v.1.34.0 [3]. We considered as “samples” the cells in clustering annotation analysis, and clusters in functional analysis.

***Functional Analyses***

Only marker genes filtered as described above were took into consideration for functional analyses (Figure 2D); furthermore marker genes were divided in positive and negative regulated ($\log_{2} FC>0 and\log_{2} FC<0$ respectively), defining UP and DOWN signatures. The p-value of the enriched pathways were calculated with gprofiler2 package [4]. The Canonical Pathways gene sets [5] derived from the KEGG pathway database, the Canonical Pathways gene sets derived from the Reactome pathway database and the Gene Ontology gene sets were taken into consideration.

***Cellular trajectory analysis***

The cellular trajectory analysis was performed using the STREAM package [6] (v.1.0) developed in Python (v.3.6.11). Only cells previously annotated as progenitors and monocytes were considered as input raw counts dataset. The variable genes were calculated and applied for branching analysis, and the Spectral Embedding (SE) was used as reduction method. The 5th state (S5) was chosen as the root node.

***SCENIC analysis***

Single-cell regulatory network inference and clustering (SCENIC)[7] analysis was performed on each cell type according to the pySCENIC (v.0.11.4) [7]. Firstly, potential TF targets based on coexpression are identified. Secondly, TF motif enrichment analysis was performed to identify the direct targets (regulons) and score the activity of the regulons (AUCell score). Results were then represented in binary considering calculated thresholds and plotted in a heatmap.

## Data analysis (GC dataset and CoV-2 dataset)

For the GC dataset, only data from time points C1, C3 and C5 were considered. For the CoV-2 dataset only data from patients between 20 and 80 years old were considered. Data from patient P02 were not available and thus not included in the analysis. Two matrices of 55,293 and 100,897 cells were obtained from the GC and CoV-2 dataset, respectively.

***RNA-data processing***

Preliminary data filtering, data integration and markers analyses were performed as for the HD dataset. Clustering was achieved by using a resolution of 0.6. In order to annotate clusters, each cell was subjected to the single-sample Gene Set Enrichment Analyses (ssGSEA) using manually curated signatures (**Tables S4-5**).

***Monocyte classification using a machine learning model***

Monocytes classification was obtained using a machine learning model developed with the caret R package [8], considering a polynomial Kernel support vector machine. The HD dataset was divided randomly in two parts: one training set (80% of the healthy monocyte dataset) and one test set (20%). 161 features were selected in order to train the model; features were obtained as the intersection of monocyte marker genes (previously defined in the HD dataset) and variable genes in the tumor dataset. The quality of the model was evaluated considering sensitivity, specificity and accuracy. It was tested in order to avoid overfitting typical errors in machine learning developmental models. Cross validation (tenfold) was performed. After accurate evaluations, the model trained in the healthy monocyte dataset was applied in both the GC dataset and the CoV-2 dataset and monocytes were classified considering features patterns. In this way, we were able to recognize the defined clusters of the healthy dataset in both the cancer patients and the CoV-2 datasets.

***Single-sample Gene Set Enrichment Analysis (ssGSEA)***

In the GC dataset, ssGSEA of the BURTON_ADIPOGENESIS_1, BILD_HRAS_ONCOGENIC_SIGNATURE and HALLMARK_TNFA_SIGNALING_VIA_NFKB pathways was performed with the packages AnnotationDbi v.1.48.0, org.Hs.eg.db v.3.10.0 qusage, and GSVA v.1.34.0. We considered as “samples” the monocyte clusters.

## Plots

Plots were performed with the following packages: Serat [9], pheatmap (lnc. https://rdrr.io/cran/pheatmap/), ggplot2 [10], and STREAM [6].

SUPPLEMENTAL REFERENCES

1. Barbie, D.A., et al., *Systematic RNA interference reveals that oncogenic KRAS-driven cancers require TBK1.* Nature, 2009. **462**(7269): p. 108-12.

2. Yaari, G., et al., *Quantitative set analysis for gene expression: a method to quantify gene set differential expression including gene-gene correlations.* Nucleic Acids Res, 2013. **41**(18): p. e170.

3. Hänzelmann, S., R. Castelo, and J. Guinney, *GSVA: gene set variation analysis for microarray and RNA-seq data.* BMC Bioinformatics, 2013. **14**: p. 7.

4. Kolberg, L., et al., *gprofiler2 -- an R package for gene list functional enrichment analysis and namespace conversion toolset g:Profiler.* F1000Res, 2020. **9**.

5. Subramanian, A., et al., *Gene set enrichment analysis: a knowledge-based approach for interpreting genome-wide expression profiles.* Proc Natl Acad Sci U S A, 2005. **102**(43): p. 15545-50.

6. Chen, H., et al., *Single-cell trajectories reconstruction, exploration and mapping of omics data with STREAM.* Nat Commun, 2019. **10**(1): p. 1903.

7. Aibar, S., et al., *SCENIC: single-cell regulatory network inference and clustering.* Nat Methods, 2017. **14**(11): p. 1083-1086.

8. Kuhn, M., *Building Predictive Models in R Using the caret Package.* 2008:  J*ournal of Statistical Software*. p. 1–26.

9. Hao, Y., et al., *Integrated analysis of multimodal single-cell data.* Cell, 2021. **184**(13): p. 3573-3587.e29.

10. Wickham, H., *ggplot2: Elegant Graphics for Data Analysis*. 2016: Springer-Verlag New York.

SUPPLEMENTAL TABLES

## Table S1. List of fluorophore-conjugated antibodies used in this study

| **Marker** | **Clone** | **Conjugate** | **Manufacturer** | **Titer (ul*)** |
| --- | --- | --- | --- | --- |
| CD45 | HI30 | BV421 | BioLegend | 2.50 |
| CD3 | UCHT1 | BV570 | BioLegend | 1.25 |
| CD19 | HIB19 | BV570 | BioLegend | 1.25 |
| CD56 | 51H11 | BV570 | Biolegend | 2.50 |
| HLA-DR | L243 | APC-Cy7 | BD Biosciences | 1.25 |
| CD66b | G10F5 | AF700 | BioLegend | 2.50 |
| CD14 | M5E2 | AF488 | BD Biosciences | 1.25 |
| CD16 | 3G8 | PerCP/Cy5.5 | BioLegend | 2.50 |

| *staining in 100 ul. |
| --- |

## Table S2. Cell Ranger mapping statistics

| **Sample** | **Estimated number of cells** | **Mean reads per cell** | **Mean genes per cell** | **Total genes detected** |
| --- | --- | --- | --- | --- |
| s01 | 7,746 | 64,048 | 2,293 | 22,673 |
| s02 | 7,186 | 68,804 | 2,270 | 22,707 |
| s03 | 7,785 | 65,220 | 2,270 | 22,668 |
| s04 | 5,932 | 65,897 | 2,156 | 21,686 |
| s05 | 6,986 | 71,024 | 2,342 | 22,970 |

## Table S3. List of gene signatures used to identify cell types in the HD dataset

| **CD14+ mono** | **CD16+ mono** | **DCs** | **progenitors** | **NK cells** | **B cells** |
| --- | --- | --- | --- | --- | --- |
| CD14 | FCGR3A | ID2 | KIT | NKG7 | BLK |
| VCAN | LST1 | IRF4 | CD34 | GNLY | BTK |
| S100A8 | AIF1 | FCER1A | FLT3 | GZMA | CD19 |
| S100A9 | IFITM3 | CLEC4C | GATA1 | GZMH | MS4A1 |
| S100A12 | SERPINA1 | IL3RA | GATA2 | GZMM | CD79A |
| FCN1 | MTSS1 | CD1C | MS4A3 | KLRD1 | IGHM |
| LYZ | TCF7L2 | IRF8 | SOX4 | NCR3 | CD22 |
| RNASE2 | CSF1R | CLEC10A | HOXA9 | KLRF1 | FCRL2 |
| CD36 | SIGLEC10 | CD74 | HOXA10 | CTSW | CD24 |
| NCF1 | RHOC | HLA-DQA1 | CYTL1 | IL2RB |  |
|  |  | HLA-DPB1 |  | KLRB1 |  |
|  |  | HLA-DPA1 |  | PRF1 |  |
|  |  | HLA-DQB1 |  |  |  |
|  |  | HLA-DRB1 |  |  |  |
|  |  | HLA-DMA |  |  |  |

## Table S4. List of gene signatures used to identify cell types in the GC dataset

| **CD14+ mono** | **CD16+ mono** | **DCs** | **NK cells** | **B cells** | **T cells** | **platelets** |
| --- | --- | --- | --- | --- | --- | --- |
| CD14 | FCGR3A | ID2 | NKG7 | MS4A1 | CD3E | PPBP |
| VCAN | LST1 | IRF4 | GNLY | CD79A | CD3D | GP9 |
| S100A8 | AIF1 | FCER1A | GZMA | CD79B | CD3G | PF4V1 |
| S100A9 | IFITM3 | CLEC4C | GZMH | CD22 |  | SELP |
| S100A12 | SERPINA1 | IL3RA | GZMM | FCRL2 |  | GP6 |
| FCN1 | MTSS1 | CD1C | KLRD1 | MZB1 |  | GP1BA |
| LYZ | TCF7L2 | IRF8 | NCR3 |  |  |  |
| RNASE2 | CSF1R | CLEC10A | KLRF1 |  |  |  |
| CD36 | SIGLEC10 | CD74 | CTSW |  |  |  |
| NCF1 | RHOC | HLA-DQA1 | IL2RB |  |  |  |
|  |  | HLA-DPB1 | KLRB1 |  |  |  |
|  |  | HLA-DPA1 | PRF1 |  |  |  |
|  |  | HLA-DQB1 |  |  |  |  |
|  |  | HLA-DRB1 |  |  |  |  |
|  |  | HLA-DMA |  |  |  |  |
|  |  | CLEC9A |  |  |  |  |

## Table S5. List of gene signatures used to identify cell types in the CoV-2 dataset

| **CD14+ mono** | **CD16+ mono** | **DCs** | **NK cells** | **B cells** | **T cells** | **platelets** | **progenitors** |
| --- | --- | --- | --- | --- | --- | --- | --- |
| CD14 | FCGR3A | ID2 | NKG7 | MS4A1 | CD3E | PPBP | KIT |
| VCAN | LST1 | IRF4 | GNLY | CD79A | CD3D | GP9 | CD34 |
| S100A8 | AIF1 | FCER1A | GZMA | CD79B | CD3G | PF4V1 | FLT3 |
| S100A9 | IFITM3 | CLEC4C | GZMH | CD22 |  | SELP | GATA2 |
| S100A12 | SERPINA1 | IL3RA | GZMM | FCRL2 |  | GP6 | SOX4 |
| FCN1 | MTSS1 | CD1C | KLRD1 | MZB1 |  | GP1BA | HOXA9 |
| LYZ | TCF7L2 | IRF8 | NCR3 |  |  |  | HOXA10 |
| RNASE2 | CSF1R | CLEC10A | KLRF1 |  |  |  | CYTL1 |
| CD36 | SIGLEC10 | CD74 | CTSW |  |  |  |  |
| NCF1 | RHOC | HLA-DQA1 | IL2RB |  |  |  |  |
|  |  | HLA-DPB1 | KLRB1 |  |  |  |  |
|  |  | HLA-DPA1 | PRF1 |  |  |  |  |
|  |  | HLA-DQB1 |  |  |  |  |  |
|  |  | HLA-DRB1 |  |  |  |  |  |
|  |  | HLA-DMA |  |  |  |  |  |
|  |  | CLEC9A |  |  |  |  |  |

SUPPLEMENTAL FIGURES

FIGURE S1
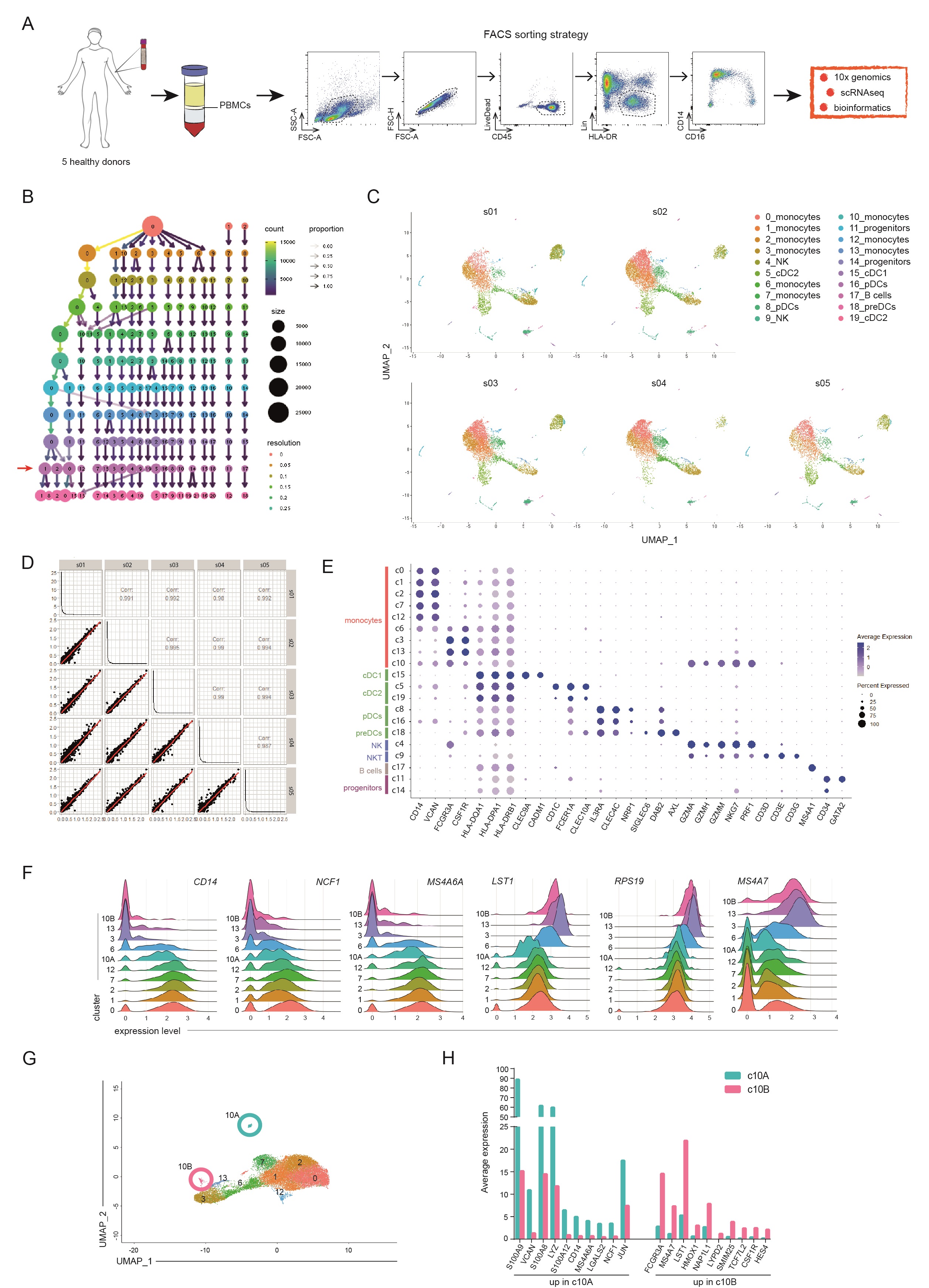


## Figure S1. Single-cell RNA sequencing of healthy peripheral blood HLA-DR+ cells identified 20 cell clusters belonging to 5 distinct cell lineages.

(**A**) Illustration of the experimental workflow and gating strategy adopted for the scRNAseq. Blood samples were collected from 5 healthy donors and PBMCs were isolated by Lympholyte density gradient. Live lineage^-^HLA-DR^+^ cells were FACS-sorted and loaded for 10X Genomics platform based scRNA-seq. lin = lineage (CD3, CD19, CD56 cells) (**B**) Plot of clustering tree at different resolutions (from 0 to 0.5; each level is increased by 0.05). Relationship between clusters at different resolutions and proportion and count of cells within each cluster are shown. Clusters at 0.45 resolution value are pointed out by a red arrow. (**C**) UMAP projections showing all the cell clusters identified individually in each donor (s01-05). Clusters are colored according to cluster designation. (**D**) Scatter plot matrix representing Pearson’s correlation coefficients of gene expression values in each cell between pairwise donors (s01-05). Rows and columns represent samples. Abscissa and Ordinate axes show the logarithmic gene expression. Each dot represents a gene. s01-05, sample 01-05 (each sample corresponds to one donor). (**E**) Dot-plot showing the expression of key genes adopted for manual cell cluster annotation. Gene expression is colored coded from gray (lower) to blue (higher); circle size indicates the fraction of cells expressing the gene. (**F**) Ridge plot showing the expression levels of typical genes highly expressed in classical (top) and non-classical (bottom) monocytes. Only monocyte subsets are shown and clusters are coloured according to cluster designation. (**G**) UMAP projection of monocytes showing the distribution of the two sub-clusters c10A (jade-green circle) and c10B (pink circle) identified by graph-based clustering. Clusters are numbered as in Fig. 1B. Each dot represents an individual cell. (**H**) Bar plot showing the top-20 DEGs between cluster c10A and c10B. 10 genes upregulated in c10A (left) and 10 genes upregulated in c10B (right) are shown.

**
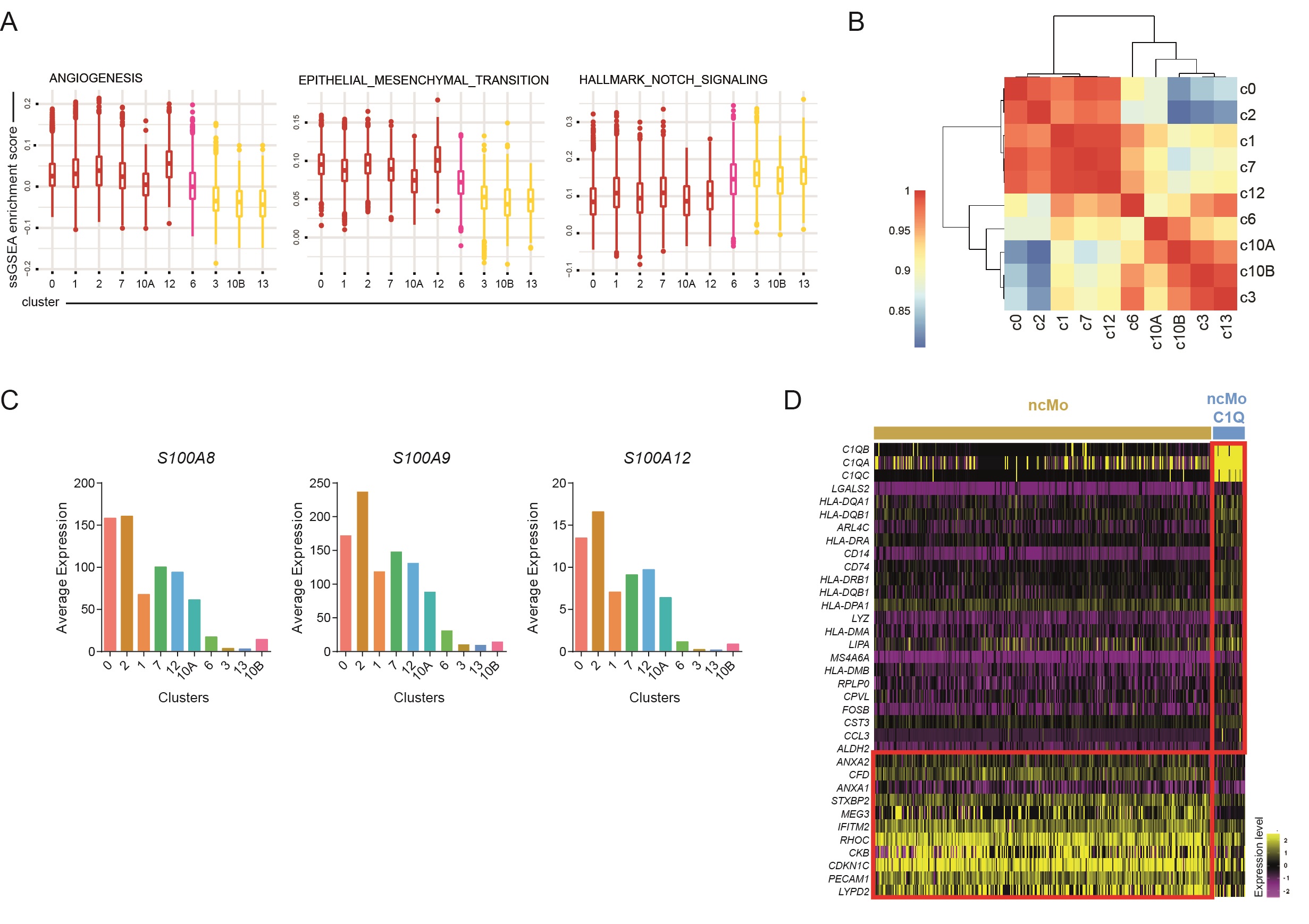
FIGURE S2**

## Figure S2. Characterization of transcriptional heterogeneity among human circulating monocytes in homeostatic conditions.

(**A**) Boxplot showing the normalized enrichment score (ssGSEA) of selected Hallmark signatures found equally enriched in clusters belonging to the same monocyte macro-group (classical and non-classical monocytes). Color code: dark red, classical monocytes; pink, intermediate monocytes; yellow, non-classical monocytes. (**B**) Heatmap representing Person’s correlation between the 10 subtypes of monocytes. Pearson’s coefficient values are colored-coded from blue (lower) to red (higher). (**C**) Bar plots showing the average expression of *S100A8/9/12* in each cluster of monocytes. (**D**) Single-cell gene expression heatmap showing significant differentially expressing genes (
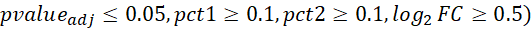
 among non-classical monocyte cell subsets (c3 and c13). Gene expression is colored-coded from purple (lower) to yellow (higher); gene expression level is scaled by row.

**FIGURE S3**


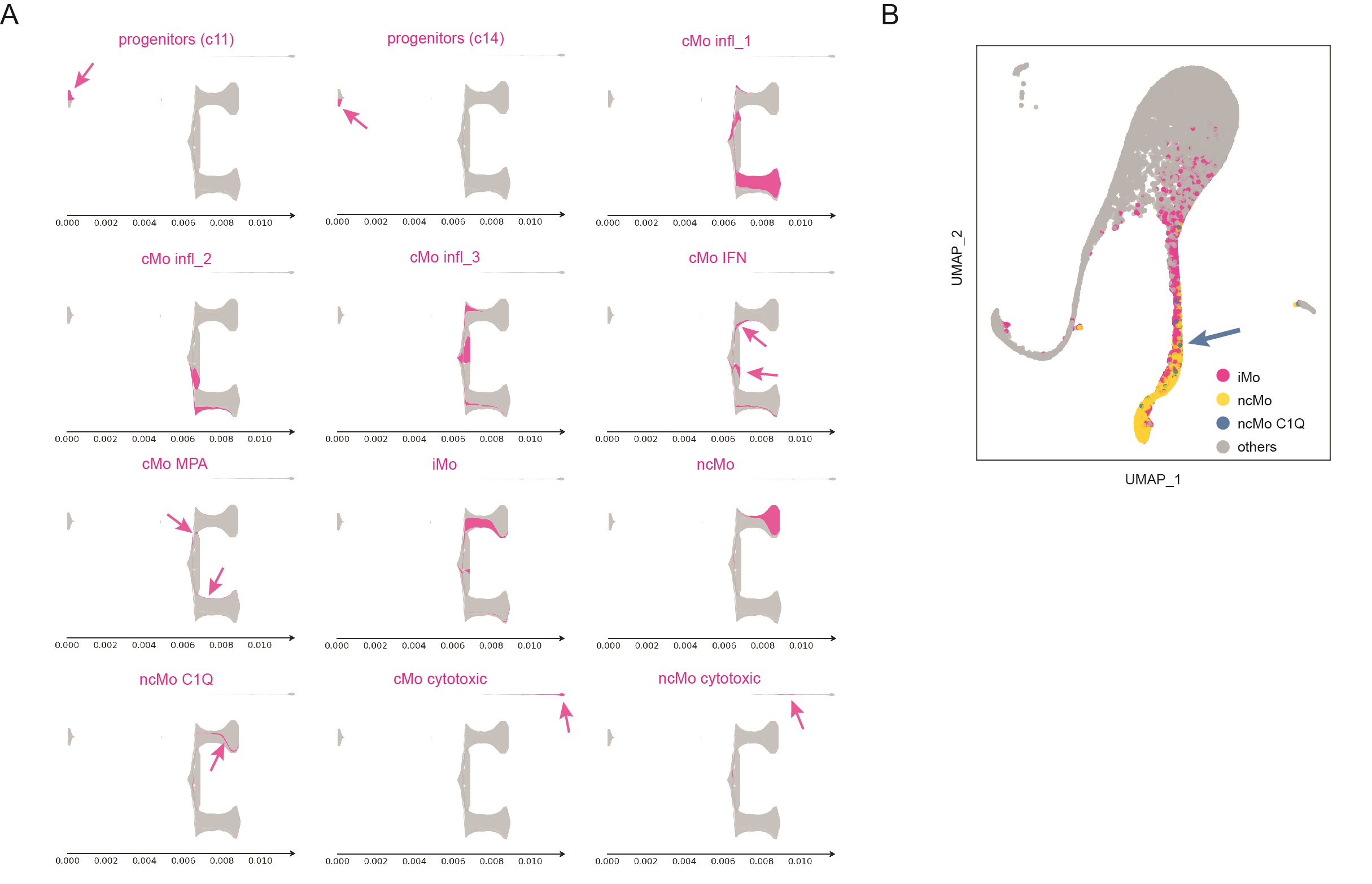


## Figure S3. Monocyte subsets developmental relationship.

(**A**) Stream plots visualization of inferred developmental trajectory of myeloid progenitors and monocytes by STREAM. Each plot shows in pink one single cluster from the scRNA-seq. (**B**) UMAP visualization of inferred developmental trajectory of myeloid progenitors and monocytes by STREAM highlighting intermediate monocytes and non-classical monocytes from cluster 3 and 13.

**FIGURE S4**

**
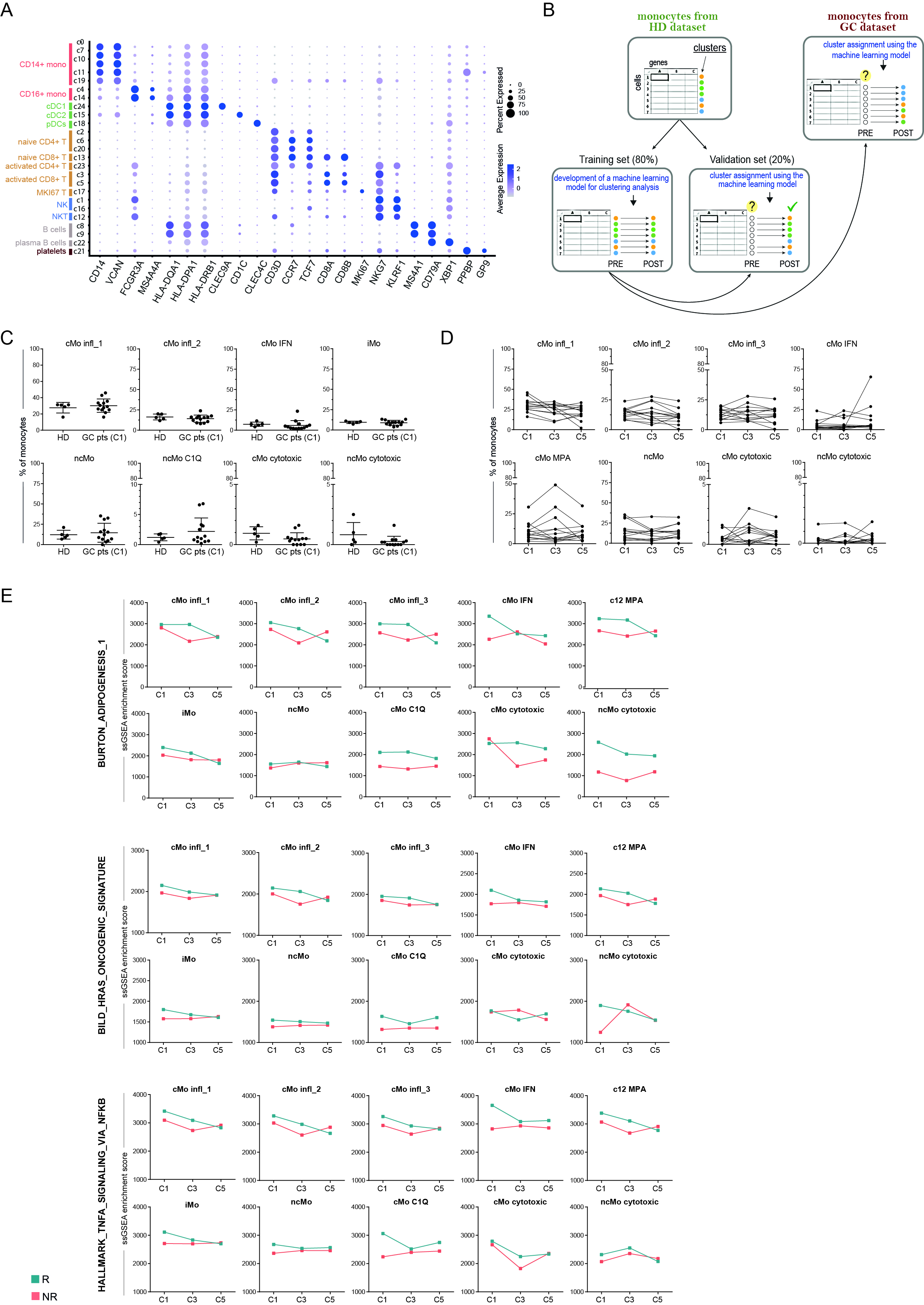
**

## Figure S4. Monocyte subsets in cancer immunotherapy.

(**A**) Dot-plot showing the expression of key genes adopted for manual cell cluster annotation of the GC dataset. Gene expression is colored coded from gray (lower) to blue (higher); circle size indicates the fraction of cells expressing the gene. (**B**) Schematic illustration of the machine learning classifier developed using information from healthy donors and then adopted for the re-clusterization of monocytes from GC tps. (**C**) Graph showing the proportion of monocyte subsets over the total monocyte population in healthy donors and in GC pts before treatment (time point C1). Statistical significance was determined by the Mann-Whitney test. Percentages of cMo_infl_3 and cMo_MPA are illustrated in Figure 4D. (**D**) Graph showing the fraction of each monocyte subsets in GC pts during therapy. Each line represents a patient percentage trend along the three therapy cycle steps. Statistical significance was determined by the Friedman test followed by Dunnett’s multiple comparison test. Percentage of iMo and nc_Mo C1Q are illustrated in Figure 4E. (**E**) Graph representing the trend of pathway enrichment of BURTON_ADIPOGENESIS_1, BILD_HRAS_ONCOGENIC_SIGNATURE and ALLMARK_TNFASIGNALING_VIA_NFKB pathways in GC pts responders (green-blue) and non-responders (red) during therapy.

C1, cycle 1 = baseline; C3, cycle 3 = chemotherapy mFOLOFX6 regimen; C5, cycle 5 = chemotherapy + anti–PD-1 immunotherapy. GC pts, gastrointestinal cancer patients. NS, non-responder patients; R = responder patients.

**FIGURE S5**


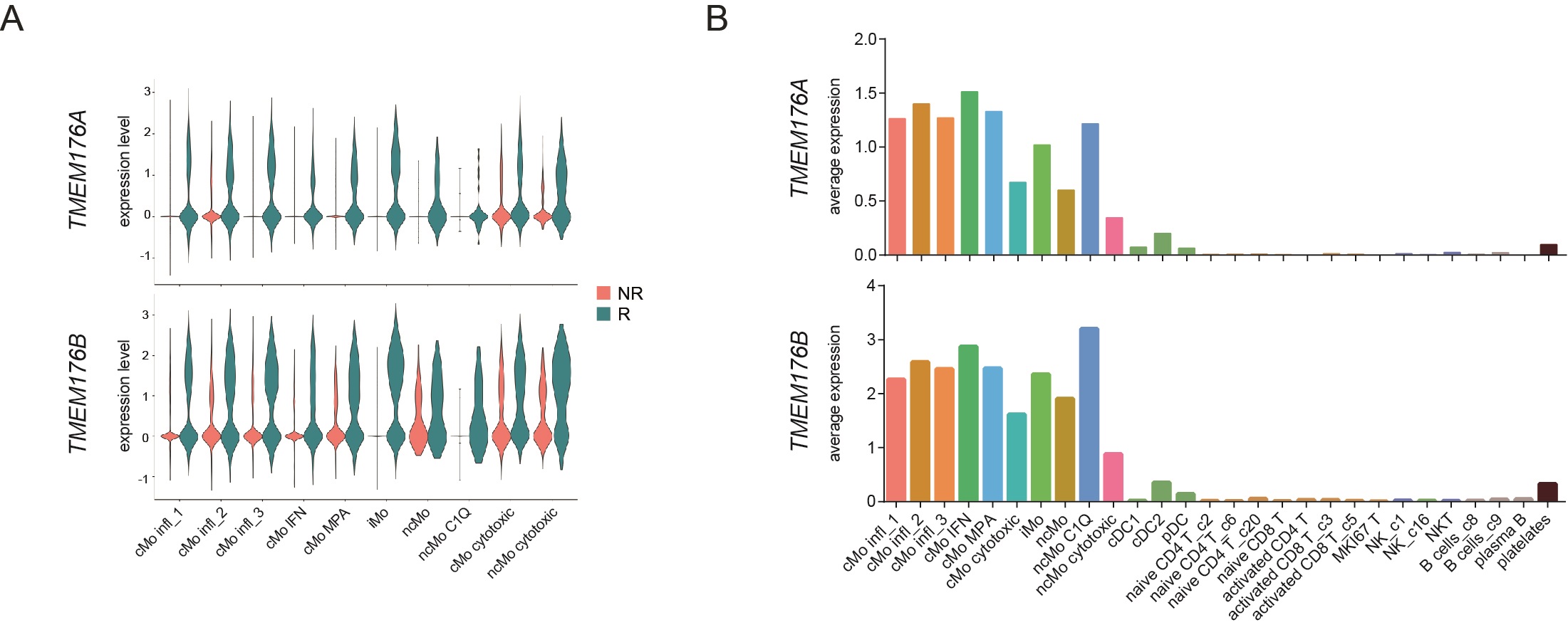


## Figure S5. TMEM176A and TMEM176B expression in monocytes.

(**A**) Violin plot showing the expression distribution of *TMEM176A* (up) and *TMEM176B* (bottom) genes in human circulating monocyte populations from responders (green-blue) and non-responders (red) in the GC dataset. (**B**) Bar plots showing the expression of *TMEM176A* (up) and *TMEM176B* (bottom) in each cell cluster of the GC dataset. Clusters are colored according to their lineage.

NS, non-responder patients; R = responder patients.

**FIGURE S6**

**
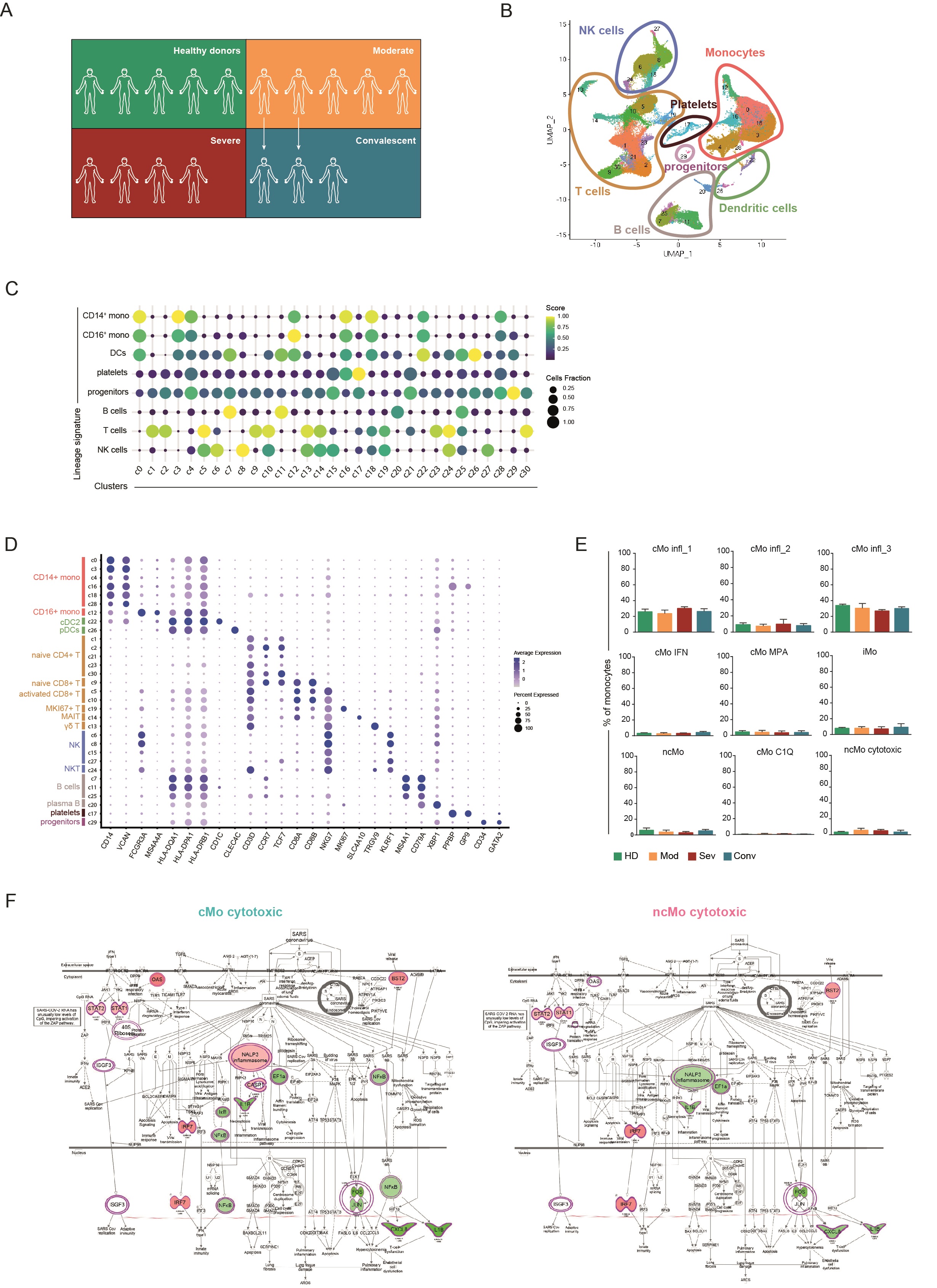
**

## Figure S6. Monocyte subsets in COVID-19 patients

(**A**) Schematic illustration of the study design adopted in the study of Zhang and colleagues (Mod. from Zhang et al., Nat. Immunol., 2020). PBMCs were isolated from healthy donors and from patients with COVID-19 across three conditions. Single cell RNA sequencing was performed on the PBMCs samples using 10X Genomics technology and sequenced on an Illumina Novaseq6000 sequencer with a paired-end 150-bp reading strategy. (**B**) UMAP projection of PBMCs from the CoV-2 (n = 100,897) dataset showing 31 clusters belonging to 7 major immune cell subsets. Clusters are numbered according to their size, from the largest (cluster 0) to the smallest (cluster 30). Each dot represents an individual cell. (**C**) Dot-plot showing annotated immune cells by lineage signatures (genes belonging to each signature are listed in Table S5). ssGSEA score-based signature expression is colored-coded from blue (lower) to yellow (higher); circle size indicates the fraction of cells expressing the signature. (**D**Dot-plot showing the expression of key genes adopted for manual cell cluster annotation of Cov-2 dataset. Gene expression is colored coded from gray (lower) to blue (higher); circle size indicates the fraction of cells expressing the gene. (**E**) Graph bar showing the fraction of each monocyte subsets over the total monocyte population across four conditions. Statistical significance was determined by Kruskal-Wallis one-way ANOVA test followed by Dunn's multiple comparisons test. Percentage of cMo cytotoxic is illustrated in Figure 5C. (**F**) Overview of the Coronavirus Pathogenesis Pathway from QIAGEN® Ingenuity® Pathway Analysis (IPA®) in cMo cytotoxic (left) and ncMo cytotoxic (right) from severe COVID-19 pts vs healthy donors. Pink-colored outline: dataset molecule in the pathway; pink/red color: up-regulation or increased phosphorylation; green color: down-regulation or decreased phosphorylation; white color: molecule not present in the dataset; double-borders: group or complex.

**FIGURE S7**


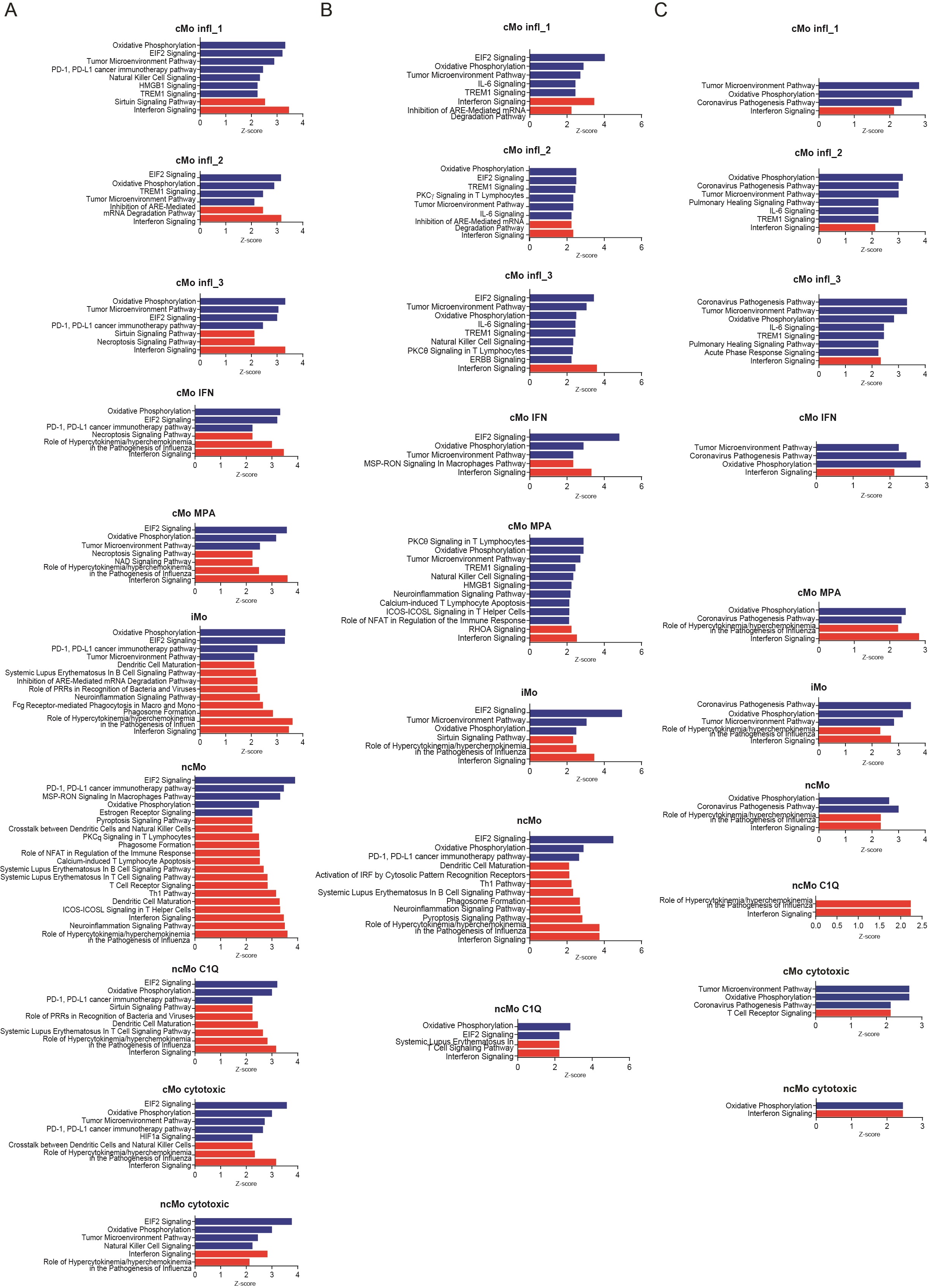


## Figure S7. Ingenuity pathway analysis of the CoV-2 dataset.

(**A-C**) Ingenuity Pathway Analysis of the DEGs between monocytes from moderate COVID-19 pts and healthy donors (**A**); severe COVID-19 pts and healthy donors (**B**) or convalescent COVID-19 pts and healthy donors (**C**). Only pathways that were significantly (-log_10_(p-val) > 1.3) upregulated (z score > 2, in red) or downregulated (z score < -2, in blue) are shown. Analysis of cytotoxic monocytes in the comparison between severe pts vs healthy donors are shown in Fig. 5E.
